# A double helical motif in OCIAD2 is essential for its localization, interactions and STAT3 activation

**DOI:** 10.1038/s41598-018-25667-3

**Published:** 2018-05-09

**Authors:** Saloni Sinha, Venkata Anudeep Bheemsetty, Maneesha S. Inamdar

**Affiliations:** 1 Jawaharlal Nehru Centre for Advanced Scientific Research, Jakkur, Bangalore, 560064 India; 2 Institute for Stem Cell Biology and Regenerative Medicine, GKVK, Bellary Road, Bangalore, 560065 India

## Abstract

The Ovarian Carcinoma Immunoreactive Antigen domain (OCIAD) - containing proteins OCIAD1/Asrij and OCIAD2, are implicated in several cancers and neurodegenerative diseases. While Asrij has a conserved role in facilitating STAT3 activation for JAK/STAT signaling, the expression and function of OCIAD2 in non-cancerous contexts remains unknown. Here, we report that *ociad2* neighbors *ociad1/asrij* in most vertebrate genomes, and the two genes likely arose by tandem gene duplication, probably somewhere between the Ordovician and Silurian eras. We show that *ociad2* expression is higher in the mouse kidney, liver and brain relative to other tissues. OCIAD2 localizes to early endosomes and mitochondria, and interacts with Asrij and STAT3. Knockdown and overexpression studies showed that OCIAD2 is essential for STAT3 activation and cell migration, which could contribute to its role in tumor metastasis. Structure prediction programs, protein disruption studies, biochemical and functional assays revealed a double helical motif in the OCIA domain that is necessary and sufficient for its localization, interactions and STAT3 activation. Given the importance of JAK/STAT signaling in development and disease, our studies shed light on the evolution and conserved function of the OCIA domain in regulating this pathway and will be critical for understanding this clinically important protein family.

## Introduction

OCIAD1 and OCIAD2 are human cancer-related proteins implicated in ovarian^1^, thyroid^2^ and lung cancers^3^, and in various hematological neoplasms^4,5^ including multiple myeloma^6^ and neutrophilia^7^. Their names derive from the first report on OCIAD1 (Ovarian Carcinoma Immunoreactive Antigen domain-containing protein 1), which was found in ascites fluid of patients with metastatic ovarian cancer and mapped to chromosome 4p11^8^. Subsequently, a smaller human protein sharing homology with the N-terminal region of OCIAD1 was identified and designated OCIAD2. While the developmental expression and cellular function of Asrij, the mouse ortholog of human OCIAD1 is reported^9^, there is limited information on the normal expression and localization of human OCIAD2 or its orthologs.

Asrij localizes to endosomes^10^ and mitochondria^11^ and has key conserved roles in the maintenance of stemness in *Drosophila* hematopoiesis^12^, as well as in mouse embryonic stem cell pluripotency^9^. Moreover, Asrij regulates blood cell homeostasis in *Drosophila* and its absence causes fly leukemia^12^. Asrij has a conserved role in regulating the JAK/STAT and Notch signaling pathways^9,12^.

Although OCIAD2 expression varies among different cancers, its precise function remains unknown - while high levels of OCIAD2 are reported in ovarian mucinous tumors^1^ and lung carcinomas^3,13^; significantly reduced OCIAD2 expression is reported in liver and gastric carcinomas^14^, glioblastomas^15^ and chronic lymphocytic leukemia^16^. Further, loss of OCIAD2 function promotes cancer progression by increasing activation of the PI3K/Akt pathway^17^. Moreover, the human OCIAD proteins are known to localize to lipid-rafts^18,19^ and have been proposed to be involved in the amyloidogenic processing of proteins associated with proteinopathies such as Alzheimer’s^18,19^ and Parkinson’s disease^20^.

In this study, we explore the origin, evolution and function of *ociad2*. We report that the vertebrate OCIAD family members are genomic neighbors that possibly arose by a tandem gene duplication event in the last common ancestor of jawed vertebrates. Further by *in silico*, *in situ* and biochemical approaches, we show that the two OCIAD proteins interact via a double helical region in the OCIA domain. In non-cancerous cells, OCIAD2 also interacts with and regulates STAT3 activation and cell migration, which is important in several developmental and immune processes as well as cancer. Our studies will help decipher the role and regulation of the OCIAD family proteins in various normal and pathological contexts.

## Results

### *ociad2* is located next to *ociad1*/*asrij* and encodes an OCIAD family protein

**Figure 1:**
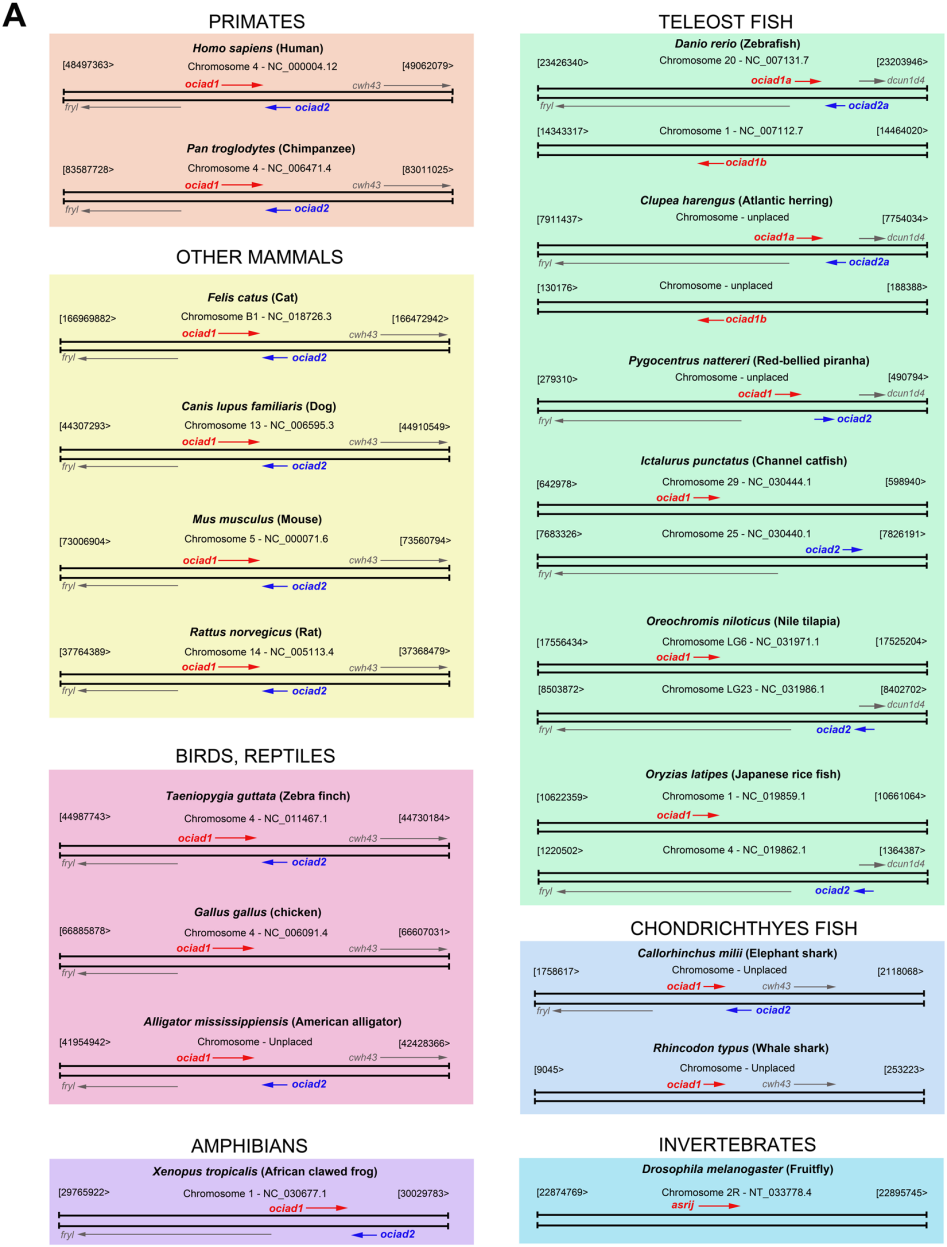
Comparison of the genomic organization for *ociad1* and *ociad2* across different species. Schematics depict genomic region encompassing *ociad1* (red) and *ociad2* (blue) and flanking genes *fryl*, *cwh43* or *dcun1d4* as indicated. Chromosome numbers and position of genes on sense or antisense strand are indicated. Direction of arrows indicates direction of transcription. Phyla are grouped into colored boxes.

*ociad1*/*asrij* is conserved in vertebrates and invertebrates, and has important functions in development and disease. Since the normal function of OCIAD1 in human is not known, we searched for similar proteins that may suggest its possible function. Querying the NCBI genome database by a BLASTp analysis (https://blast.ncbi.nlm.nih.gov/Blast.cgi?PAGE=Proteins) revealed a shorter protein OCIAD2 of 154 amino acids in mouse and human, with 36.36% sequence identity to the OCIA domain and 14.17% sequence identity in the non-domain region of OCIAD1. *In silico* analysis mapped the corresponding mouse gene to chromosome 5 at 38.54 cM (73322199–73341028 bp), which is next to *asrij* (38.44 cM, 73292784–73314069 bp) and is transcribed from the antisense strand in the opposite direction (Fig. 1A). The human protein OCIAD2 also mapped to a gene neighboring *ociad1* on chromosome 4p11. Detailed *in silico* analysis showed that the genes coding for *ociad1* and *ociad2* are neighbors, located in the same position and relative orientation (tail-to-tail) in mammals, birds, reptiles, amphibians and fish [exceptions: chicken (*Gallus gallus*), spotted gar (*Lepisosteus oculatus*, not shown) and whale shark (*Rhincodon typus*), where *ociad2* is absent and red-bellied piranha (*Pygocentrus nattereri*), where *ociad1* and *ociad2* have a tail-to-head orientation] (Fig. 1A). Notably, we found that *ociad1* and *ociad2* are neighbors only in some fish belonging to the Actinopterygii (*Danio rerio*, *Clupea harengus*, *Pygocentrus nattereri*) and Chondrichthyes (*Callorhinchus milii*) classes, whereas the others (*Ictalurus punctatus*, *Oreochromis niloticus* and *Oryzias latipes*) had *ociad1* and *ociad2* on different chromosomes (Fig. 1A). Interestingly, apart from *ociad1* and *ociad2* (referred to as *ociad1a* and *ociad2a* in Fig. 1A), some members of the teleost fish category such as *Danio rerio* and *Clupea harengus* also had an additional *ociad1*-like gene (referred to as *ociad1b* in Fig. 1A; Gene IDs: 553528, 105903538).

Although we could not find *ociad2* in whale shark (*Rhincodon typus*), it is noteworthy that this species has a non-coding RNA for *ociad2*, that seems to have been missed by the automatic annotation procedures. In this regard, a tblastn (https://blast.ncbi.nlm.nih.gov/Blast.cgi?PROGRAM=tblastn&PAGE_TYPE=BlastSearch&LINK_LOC=blasthome) search using elephant shark (*Callorhinchus milii*) OCIAD2 (Accession ID: XP_007890924.1) against the genome of whale shark (*Rhincodon typus*), revealed the presence of an uncharacterized non-coding RNA (LOC109914671, Accession ID: XR_002258473.1), present in a similar orientation, as expected for *ociad2*. Moreover, alignment of different reading frames of the elephant shark OCIAD2 protein and the translated sequence of the LOC109914671 non-coding RNA revealed overlap of two frames with high identity values (57%, 56%) and aided in the identification of several conserved exon stretches. Thus, this suggests that the arrangement of the OCIAD family of genes is very similar across all the vertebrate species.

Analysis of gene synteny across different species helped in identification of conserved flanking genes (*fryl* and *cwh43*) in the vicinity of *ociad1* and *ociad2* in mammals, birds and reptiles (Fig. 1A). Although members of the frog and teleost fish lineages lack *cwh43*, we found synteny with the gene next to *cwh43*, namely, *dcun1d4*, to be conserved (Fig. 1A). These results indicate that the gene synteny of *ociad1* and *ociad2* is conserved and strongly suggests that this particular genomic arrangement is ancestral to all vertebrate genomes analyzed here.

**Figure 2:**
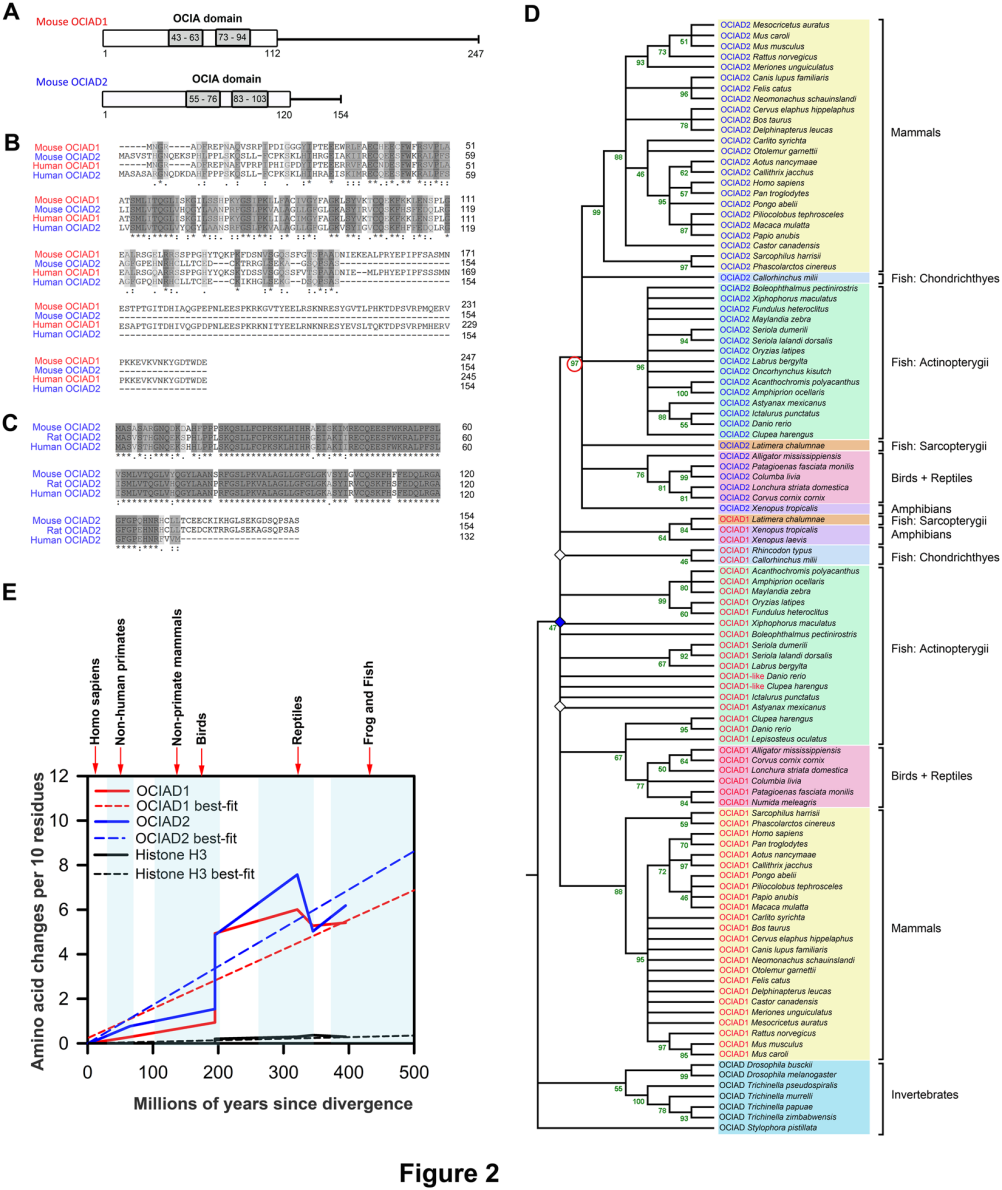
Phylogenetic analysis of the OCIAD proteins. (**A**) Schematic representation of the mouse OCIAD1/Asrij and OCIAD2 protein organization. (**B**) Multiple sequence alignment of OCIAD1/Asrij and OCIAD2 protein sequences (using MUSCLE) from mouse and human showing conserved regions. Dark shading shows amino acids identical in all sequences and light shading indicates similar amino acids. Numbers at the end of each sequence indicate amino acid positions. (**C**) Multiple sequence alignment of the predicted mouse, rat and human OCIAD2 open reading frames (using MUSCLE). (**D**) Evolutionary history of the OCIA domain proteins. The evolutionary history was inferred using the Maximum Likelihood method (see Methods). The tree with the highest log likelihood is shown. The percentage of replicate trees in which the associated taxa clustered together in the bootstrap test (100 replicates) are shown next to the branches, in green. The tree is a topology only tree and nodes with a bootstrap value less than 45 have been collapsed and shown as polytomies. Our analysis involved 106 amino acid sequences from 58 unique species. All positions containing gaps and missing data were eliminated, leaving 93 positions in the final dataset. Gene duplications are inferred using the method described in^23^. Three gene duplication events (diamonds) were identified in the tree. Open diamonds mark duplication nodes with low bootstrap values and blue diamond marks duplication node with highest bootstrap value (47). Given that there is only one copy of OCIA-domain containing proteins in invertebrates, these have been referred to as OCIAD. The extra copy of OCIAD1 protein present in *Danio rerio* and *Clupea harengus* has been listed as OCIAD1-like protein in the tree. (**E**) Sequence conservation analysis of vertebrate OCIAD1 (red line) and OCIAD2 (blue line) with respect to a conserved protein Histone H3 (black line) across different taxa. The dotted lines represent best-fit whereas solid lines represent the original values.

Mapping the nucleotide positions of the start and stop sites of *ociad1* and *ociad2* showed that these genes were non-overlapping in species where they were neighbors (Fig. S1A,B). Further, comparison of protein lengths across species shows that OCIAD2 (154 aa in mouse) is a smaller protein than OCIAD1 (247 aa in mouse) with a shorter C-terminal region. (Fig. 2A). Multiple sequence alignment of the full-length OCIAD1 and OCIAD2 sequences using MUSCLE^21^ showed maximum conservation towards the N-terminal (Fig. 2B) and a high degree of similarity between OCIAD2 sequences of mouse, rat and human (Fig. 2C). Although significant homology exists among the N-terminal regions of OCIAD1 and OCIAD2 sequences across various species, the C-terminal regions are not as well-conserved (Fig. S2A).

### *ociad1 and ociad2* evolved ca 435–500 MYA via tandem gene duplication from an ancestral *ociad* gene and have comparable rates of amino acid evolution

To explore the evolutionary relationship between OCIAD family members, we collected a total of 106 protein sequences from NCBI (https://www.ncbi.nlm.nih.gov/) covering 58 unique species, that included invertebrate OCIAD and vertebrate OCIAD1 and OCIAD2 sequences (see Table S1). We used protein sequences to identify genetic events relevant to the evolution of OCIAD family members because protein sequences are thought to be more conserved than DNA sequences^22^. These protein sequences were used to create a Maximum Likelihood (ML) phylogenetic tree with a Jones-Taylor-Thornton (JTT) model of amino acid substitution where among-site evolutionary rates are modeled with discrete gamma distribution using MEGA7 (see Table S2 and Fig. 2D). A cursory glance at the tree shows that all the OCIAD, OCIAD1 and OCIAD2 protein sequences form distinct clusters. Moreover, the monophyletic grouping of all the vertebrate OCIAD2 members is clearly visible and is also supported by the ML tree (red circle, bootstrap value: 97). To understand more about the evolutionary history of the OCIAD family of proteins, the tree was then used to identify gene duplication events^23^, and we found 3 gene duplication events (Fig. 2D). To accurately infer the history of gene duplication, we collapsed nodes bearing bootstrap values lesser than 45 (open diamonds, Fig. 2D). The duplication node with the highest bootstrap value (blue diamond, bootstrap value: 47) suggests that both OCIAD1 and OCIAD2 arose by a gene duplication event from invertebrate OCIAD (Fig. 2D) that likely occurred before the divergence of bony and cartilaginous vertebrates. Based on our observation of the earliest taxon in which OCIA domain-containing proteins were found, we propose that both OCIAD1 and OCIAD2 evolved sometime between the Ordovician and Silurian eras (435–500 mya), a period that witnessed evolution of the first jawed vertebrates^24^. Thus, based on the history of divergence of the proteins from their last common ancestor, we conclude that both vertebrate OCIAD1 and OCIAD2 are co-orthologs of invertebrate OCIAD.

Further, to estimate the comparative amino acid evolutionary patterns of the OCIA domain proteins, we calculated the number of amino acid changes per 10 sites across different species of frogs and teleost fish, reptiles, birds and mammals (Fig. 2E). Our analysis shows that the rate of change of amino acid per 10 residues in OCIAD1 and OCIAD2 are similar (Fig. 2E), suggesting possible similarity in the importance of biological functions that these two proteins perform. Additionally, although OCIAD1 sequences vary substantially across taxa, earlier work has shown that OCIAD1/Asrij plays a functionally conserved role in maintenance of stemness of *Drosophila* hematopoietic cells and mouse embryonic stem cells^9^. Similar rates of amino acid change in the two proteins and the fact that vertebrate OCIAD1 and OCIAD2 are orthologs make it likely that OCIAD2 may also have highly conserved roles and possible cooperativity or redundancy with OCIAD1 in function and regulation.

### Differential expression of *ociad2* transcript and protein in tissues and cell lines

**Figure 3:**
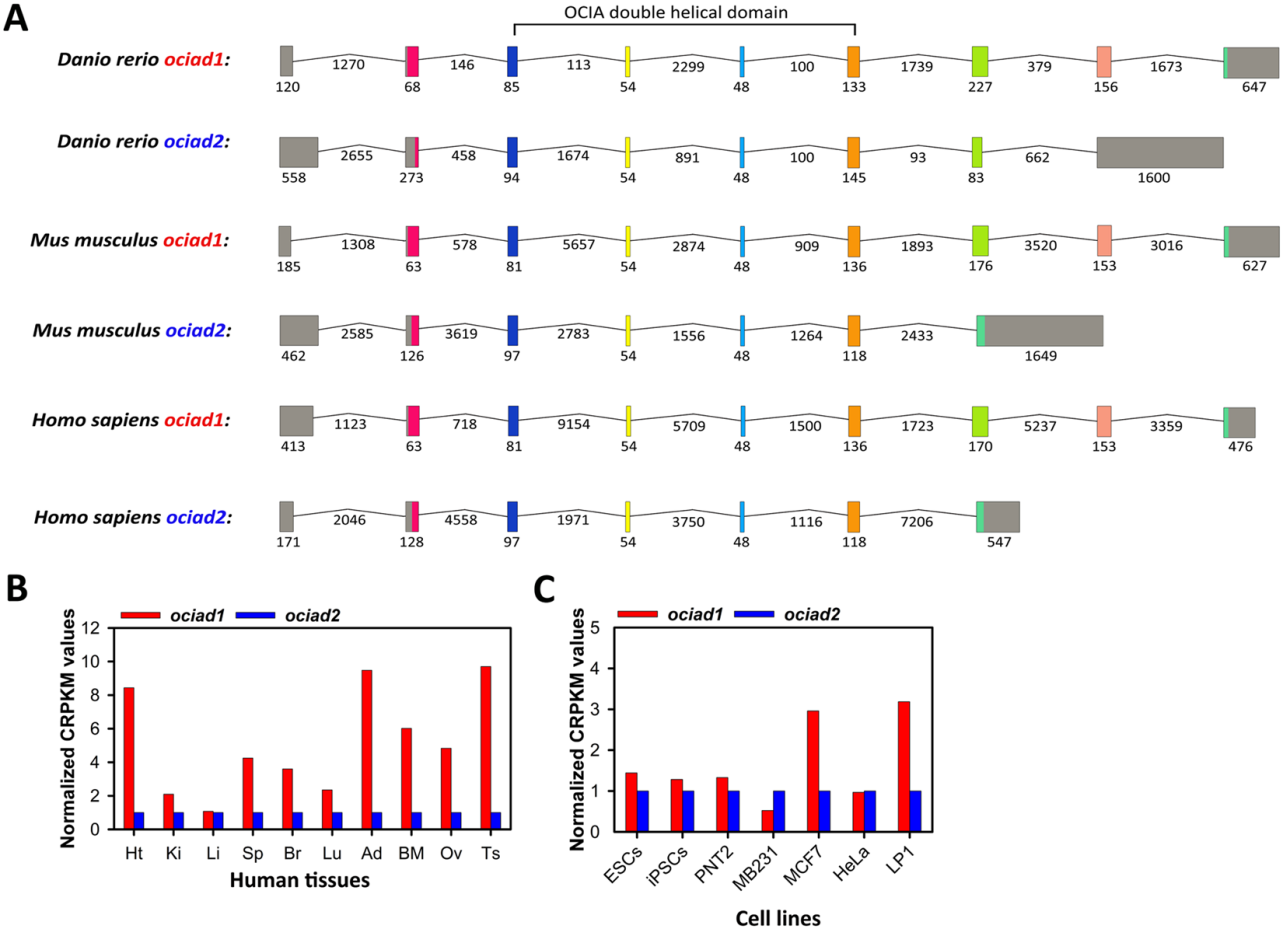
Intron-exon structure of the *ociad1/2* genes and comparison of their transcript levels in human tissues and cell lines. (**A**) Intron-exon structures of the genes coding for OCIAD1 and OCIAD2 in human, mouse and zebra fish. Intron-exon structure for the longest transcript was obtained from the Ensemble database (Accession numbers: ENST00000381473.7, ENSMUST00000031038.10, ENSDART00000103365.4, ENST00000508632.5, ENSMUST00000087195.8 and ENSDART00000164503.1). Colored boxes correspond to exons, grey-color indicates untranslated regions. Black solid lines represent introns. The size of introns and exons in nucleotides is mentioned. Introns are not drawn to scale. Exons coding for the double helical domain of OCIAD proteins have been indicated. (**B**) Comparison of transcript levels of *ociad1* and *ociad2* across different human tissues and cell lines as per data obtained from the Vertebrate Alternative Splicing and Transcription Database (VastDB, http://vastdb.crg.eu/).

To understand more about the evolutionary process that gave rise to this gene family and the evolutionary relatedness of the *ociad* genes, we compared their gene organization. Unlike *ociad1*, which comprises 9 exons in zebrafish, mouse and human, of which exon 1 and exon 9 are non-coding^10^, we found that *ociad2* is shorter and the number of exons it comprises varies across species. Mouse and human o*ciad2* contain 7 exons, while zebrafish *ociad2* contains 8 exons – in all cases exon 1 is non-coding, while the terminal exon is completely (zebrafish) or partially non-coding (mouse, human) (Fig. 3A). The presence of multiple *ociad1* isoforms lacking exons coding for the C-terminus of the protein and the shorter length of *ociad2* suggests that the latter may have lost the last exons upon gene duplication. Arrangement of the intron-exon structure of the *ociad1* and *ociad2* genes from zebrafish, mouse and human aided in identification of a particularly well-conserved region (exon 4 to exon 5) within the OCIA domain (Fig. 3A). Interestingly, *in silico* analysis using various structure prediction programs such as RaptorX (raptorx.uchicago.edu/) and Phyre^2^ (http://www.sbg.bio.ic.ac.uk/~phyre2/html/page.cgi?id=index) showed that the region ranging from exon 3 to exon 6 is indeed an important part of the *ociad1/2* genes as it codes for a double helical domain structure of the OCIAD proteins (Fig. S4).

To assess the expression of different isoforms of *ociad2* in the adult mouse, we performed semi-quantitative reverse transcriptase-polymerase chain reaction (RT-PCR) analysis on RNA isolated from various tissues using different primer combinations designed for the longest transcript of length 2510 bp (Fig. S3A). Following normalization to *gapdh*, we performed RT-PCR to detect various splice variants of *ociad2*. Full-length amplicons (F1 + R1) were expressed primarily in kidney, liver and brain; with low levels in heart, bone marrow and testis (Fig. S3B). Smaller variants encompassing exons 1 to 6 (F1 + R2), exons 1 to 5 (F1 + R3) and exons 4 to 7 (F4 + R1) were expressed in all tissues tested (Fig. S3B). Primer combination F1 + R3, revealed an isoform corresponding to 586 bp with strong expression in the brain (Fig. S3B). In addition, Sanger sequencing of the splice variants confirmed that they were *ociad2* amplicons. Protein expression analysis from these tissues by Western blot showed expression of only a 17 kDa band corresponding to the full-length OCIAD2 and no smaller isoforms were detected (Fig. S3C), though smaller isoforms have been reported for mouse (Uniprot IDs: A0A0J9YU56, A0A0J9YUI7, A0A0J9YU34 and A0A0J9YU93) and human OCIAD2 (Uniprot IDs: D6RD77, J3KPI9). In agreement with the transcript expression analysis, the full-length protein was more abundant in kidney, liver and brain than other tissues. This suggests OCIAD2 may have tissue-specific functions.

Further, to determine how *ociad2* expression varies with respect to *ociad1*, we compared their transcript levels across different human tissues and cell lines using RNA-seq datasets from VastDB^25^ (see Methods). We found that except liver, *ociad2* showed reduced transcript-level expression as compared to *ociad1* in human tissues such as heart, kidney, spleen, brain, lung, adipose, bone marrow, ovary and testis (Fig. 3B). However, in embryonic stem cells (ESCs) and induced pluripotent stem cells (iPSCs), *ociad1* and *ociad2* showed comparable levels of expression (Fig. 3C). The *ociad2* transcript is also expressed in human cell lines such as prostate epithelial cell line PNT2, breast cancer cell lines such as MB-231 and MCF-7, cervical cancer cell line (HeLa) and multiple myeloma cell line LP1 (Fig. 3C).

### OCIAD2 localizes to early endosomes and mitochondria

**Figure 4:**
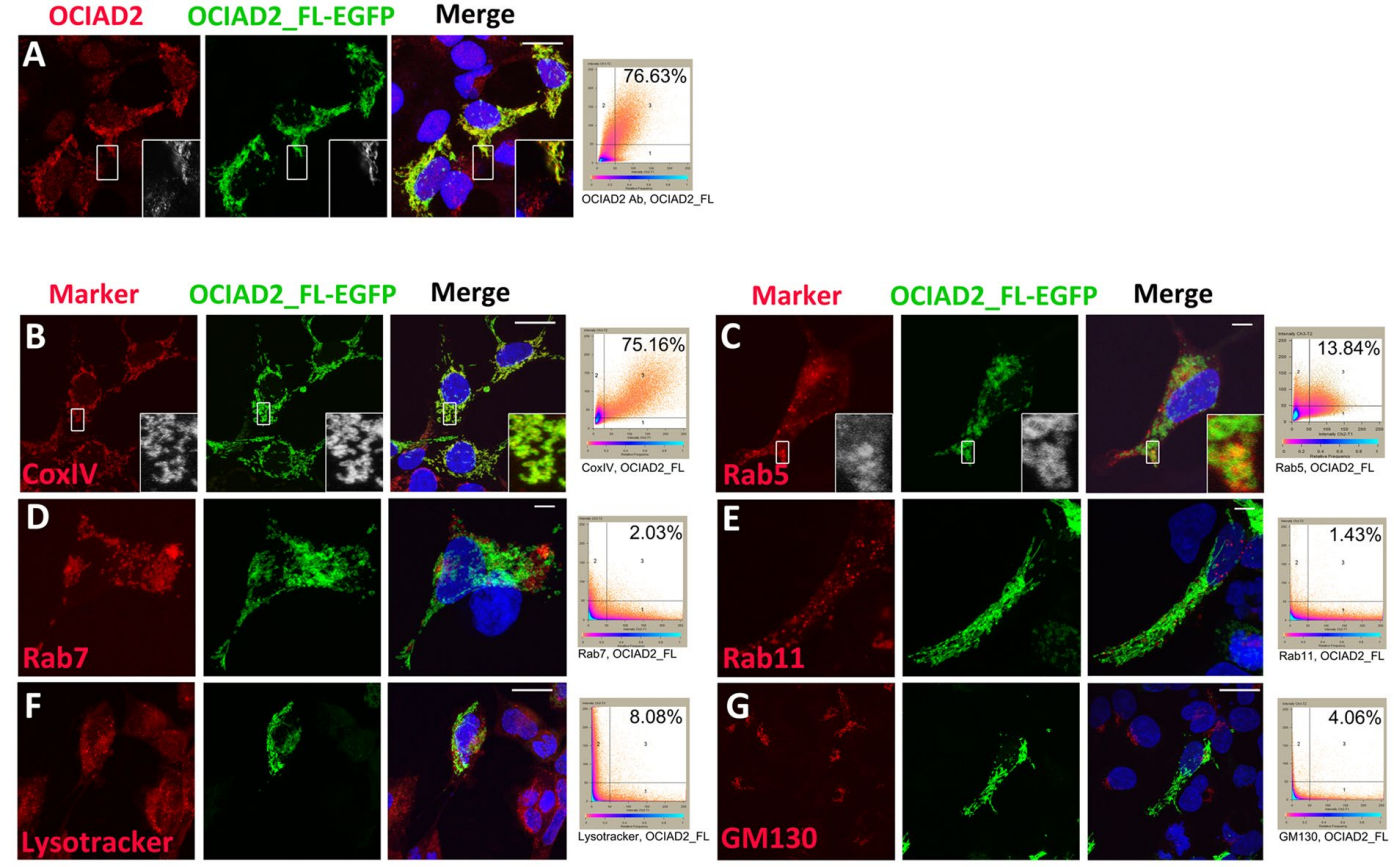
OCIAD2 localizes to endosomes and mitochondria. (**A**–**G**) Confocal images and co-localization plots of OCIAD2_FL-EGFP transfected HEK293 cells showing localization of OCIAD2 with various markers detected by immunofluorescence staining (red). (**A**) endogenous OCIAD2; (**B**) cytochrome oxidase subunit IV (CoxIV); (**C**) early endosome, Rab5; (**D**) late endosome, Rab7; (**E**) recycling endosome, Rab11; (**F**) lysosome, Lysotracker; (**G**) Golgi, GM130. Insets (A, B and C) show magnified view of the boxed region. Scale bars = 5 µm in C, D, E and 10 µm in A, B, F and G.

The 154-amino acid mouse OCIAD2 protein sequence (annotated in GenBank; Gene ID: 433904) has 82.46% and 84.41% identity with its rat and human orthologs, respectively. Analysis of the OCIAD2 protein sequence yielded little information about its structure or function, perhaps owing to the fact that the OCIA domain does not bear homology to any other known transactivation domain. To understand its putative function, we analyzed the subcellular localization of OCIAD2 using commercially available antibodies reported earlier (https://atlasantibodies.com/#!/products/OCIAD2-antibody-HPA041090/references). Immunolocalization analysis in human embryonic kidney (HEK293) cells showed puncta suggesting that OCIAD2 localizes to one or more cytosolic organelles (Fig. 4A). To further validate antibody specificity and to facilitate co-localization studies with organelle marker antibodies raised in the same host (rabbit) we generated a full-length OCIAD2-GFP expression construct (OCIAD2_FL-EGFP) (see Methods). HEK293 cells transfected with OCIAD2_FL-EGFP and stained with anti-OCIAD2 antibodies showed very high co-localization (76.63%), indicating that the reporter-tagged protein indeed reflects endogenous localization. Further, cells expressing the tagged protein showed higher antibody signal, validating antibody specificity (Fig. 4A).

While OCIAD2 expression has been studied in pathological samples, there is no report on the endogenous localization of this clinically important protein in a non-cancerous cell. The subcellular location of OCIAD2 is predicted to be endosomal as well as mitochondrial (http://www.uniprot.org/uniprot/Q56VL3, http://www.genecards.org/cgi-bin/carddisp.pl?gene=OCIAD2&keywords=human,ociad2, https://asia.ensembl.org/Homo_sapiens/Gene/Ontologies/cellular_component?db=core;g=ENSG00000145247;r=4:48885019–48906937). Moreover, proteomic studies also report localization of OCIAD2 to mitochondria^26,27^. Immuno-localization studies of the GFP-tagged full-length protein (OCIAD2_FL-EGFP) expressed in HEK293 cells (see Methods) with various organelle markers showed that a significant proportion of OCIAD2_FL-EGFP localized to cytochrome oxidase subunit IV (CoxIV) expressing mitochondria (75.16%) (Fig. 4B). In addition, a small fraction localized to Rab-5 positive early endosomes (13.84%) (Fig. 4C), but showed less than 10% localization in Rab7-positive late endosomes (2.03%) (Fig. 4D), Rab11-positive recycling endosomes (1.43%) (Fig. 4E), lysosomes marked by the Lysotracker dye (8.08%) (Fig. 4F) or Golgi body marked by GM130 (4.06%) (Fig. 4G). Thus, OCIAD2 localizes primarily to early endosomes and mitochondria.

### The OCIA domain of OCIAD2 is important for regulating its cellular localization

To elucidate the functionally important features of the OCIAD2 protein, we analyzed its primary sequence using the online tool TmPred (www.ch.embnet.org/software/TMPRED_form.html). The OCIA domain is a 128-amino acid sequence located in the N-terminal region of the OCIAD proteins. The Asrij OCIA domain is necessary and sufficient for localization and function^9^. Asrij and OCIAD2 show 36.36% homology in the domain (Fig. S2), hence we postulated that the OCIA domain of OCIAD2 may be responsible for its cellular localization, interactions and function. The OCIA domain bears two hydrophobic stretches of 21–22 amino acids each (residues 55–76 and 83–103 in mouse) separated by a conserved spacer region of about 10 amino acids. These correspond to similar stretches in Asrij (residues 43–63 and 73–94 in mouse) suggesting that OCIAD2, like Asrij, may be a membrane-associated protein (Fig. S4A). In agreement with the results obtained from hydrophobicity analysis and with the help of various structure prediction programs such as RaptorX and Phyre^2^ (see Methods), we identified a pair of helices in the central region of mouse OCIAD2 (*p* value = 9.27e-03) (Fig. S4B) located between residues lysine 41 to leucine 117 (Fig. S4C,D).

**Figure 5:**
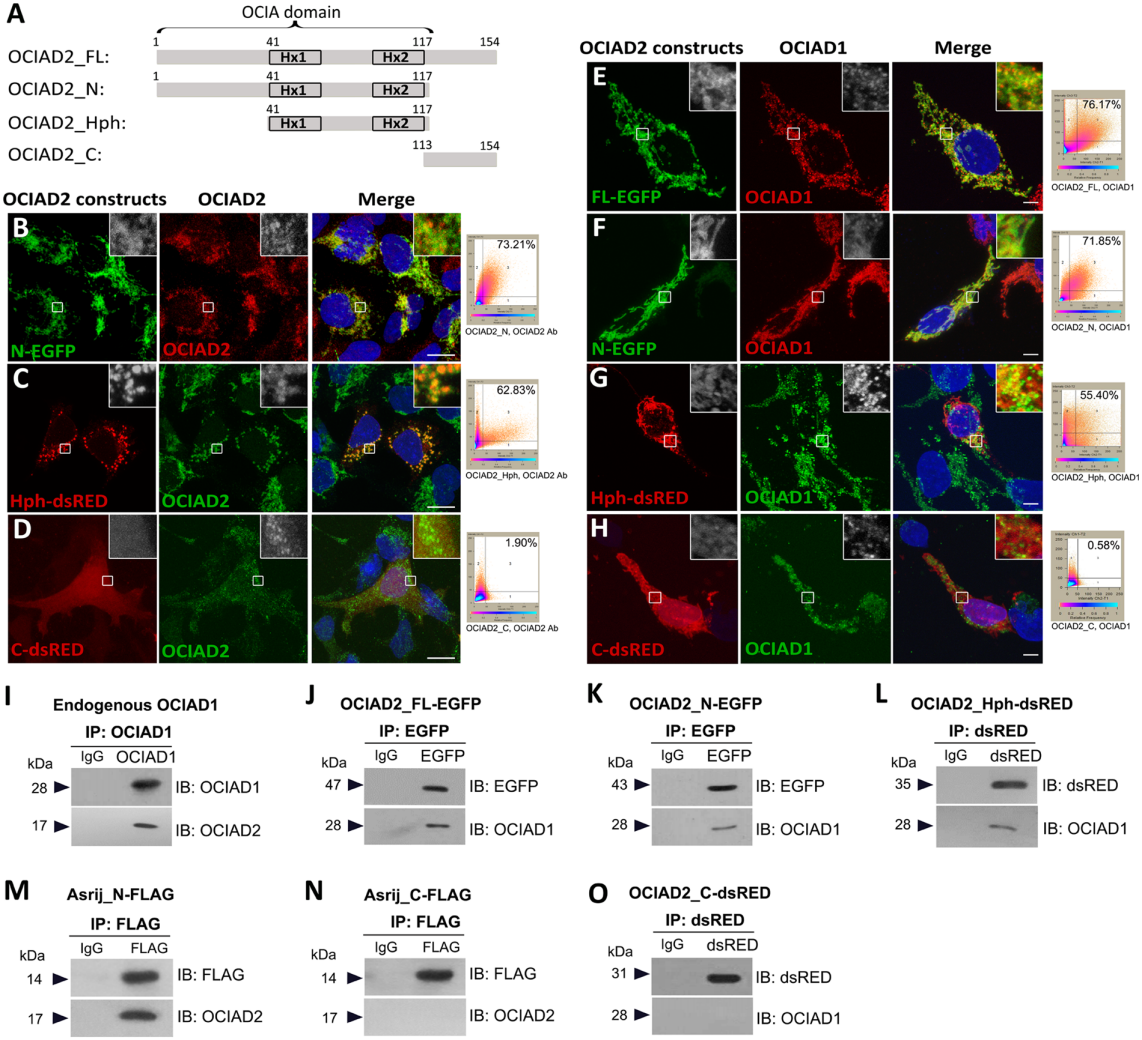
The double helical motif of OCIAD2 is necessary for interaction with OCIAD1. (**A**) Schematic representation of full-length (FL), N-terminal (N), hydrophobic region (Hph) and C-terminal (C) fragments of OCIAD2 generated for this study. Numbers indicate amino acid positions. Hx: predicted alpha helix. (**B**–**H**) Confocal images of HEK293 cells transfected with OCIAD2 reporter constructs, showing localization of ectopic OCIAD2-reporter with endogenous OCIAD2 (**B**–**D**) or endogenous OCIAD1 (**E**–**H**) as indicated, detected by fluorescence immunostaining with respective antibodies. Insets show magnified view of the boxed region. Co-localization plots are to the right of each panel. Scale bar: (**B**–**D**): 10 µm; (**E**–**H**): 5 µm. (**I**–**O**) Cell lysates from untransfected HEK293 cells (I) or those expressing various OCIAD2 (J, K, L, O) or OCIAD1/Asrij (**M**,**N**) constructs as indicated on the top of each panel, were subjected to immunoprecipitation (IP) followed by immunoblotting (IB) with antibodies as indicated. Protein size markers (in kDa) are indicated to the left. At least two independent immunoprecipitation experiments with two technical replicates each were performed and similar results were obtained. Full-length blots are presented in Supplementary Figure S6.

To assess the function of various regions of OCIAD2 protein, we generated fluorescent reporter constructs to express tagged versions of the full-length (OCIAD2_FL, 1–154 aa), N-terminal region (OCIAD2_N, 1–117 aa), conserved hydrophobic stretches that include the two helices (OCIAD2_Hph, 41–117 aa) and the C-terminal region (OCIAD2_C, 113–154 aa) of OCIAD2 (Fig. 5A) (see Methods). Transfection of constructs in HEK293 cells showed that while OCIAD2_FL-EGFP (76.63%) (Fig. 4A) and OCIAD2_N-EGFP localization overlaps largely with endogenous OCIAD2 (73.21%) (Fig. 5B), OCIAD2_Hph-dsRED shows a vesicular pattern with slightly reduced endogenous co-localization (62.83%) (Fig. 5C). In contrast, OCIAD2_C-dsRED localization was non-punctate and uniformly cytoplasmic (Fig. 5D) reminiscent of Asrij-C terminal fragment (AsrijC)^9^.

Given the conservation in sequence, we compared OCIAD2 localization with that of Asrij/OCIAD1, so that it may inform us of possible function. Co-staining cells expressing various reporter-tagged OCIAD2 fragments for Asrij showed very high co-localization index for full-length OCIAD2 (76.17%) and OCIAD2_N-EGFP (71.85%), whereas OCIAD2_Hph-dsRED (55.40%) had moderate overlap with OCIAD1 (Fig. 5E–G) and OCIAD2_C-dsRED showed no significant co-localization with OCIAD1 (Fig. 5H). Thus, the OCIA domain of OCIAD2 bearing the 77-aa long central region with two alpha helices is sufficient for its correct subcellular localization.

### The OCIA domain of OCIAD2 interacts with OCIAD1

The overlap in sequence and expression of OCIAD1 and OCIAD2 led us to speculate that they may interact. Immuno-pulldown experiments in HEK293 cells (see Methods) showed that endogenous OCIAD interacts with endogenous OCIAD2 (Fig. 5I). To identify the protein regions essential for interaction, we overexpressed the various tagged constructs of OCIAD2 in HEK293 cells and tested interactions by immunoprecipitation from cell lysates (see Methods). OCIAD2_FL-EGFP, OCIAD2_N-EGFP and OCIAD2_Hph-dsRed could all co-immunoprecipitate endogenous OCIAD1 (Fig. 5J–L). Conversely, endogenous OCIAD1 (Fig. 5I) or FLAG-tagged Asrij-N (Fig. 5M) could also immunoprecipitate endogenous OCIAD2. Asrij-N terminal fragment (1–132 aa) bearing the OCIA domain is known to interact with other proteins (STAT3, ARF1) and is the functional domain whereas Asrij-C has a dominant negative effect^9,28^. We found that AsrijC-FLAG (133–257 aa) could not immunoprecipitate endogenous OCIAD2 (Fig. 5N). Similarly, OCIAD2_C-dsRed could not immunoprecipitate endogenous OCIAD1 (Fig. 5O). This indicates that the C terminal fragments of both proteins do not interact, suggesting that the two proteins oligomerize via their OCIA domain. Further, the double helical region of OCIAD2 is sufficient for interaction with OCIAD1/Asrij.

### The double helical motif of OCIAD2 is necessary to promote STAT3 activation

**Figure 6:**
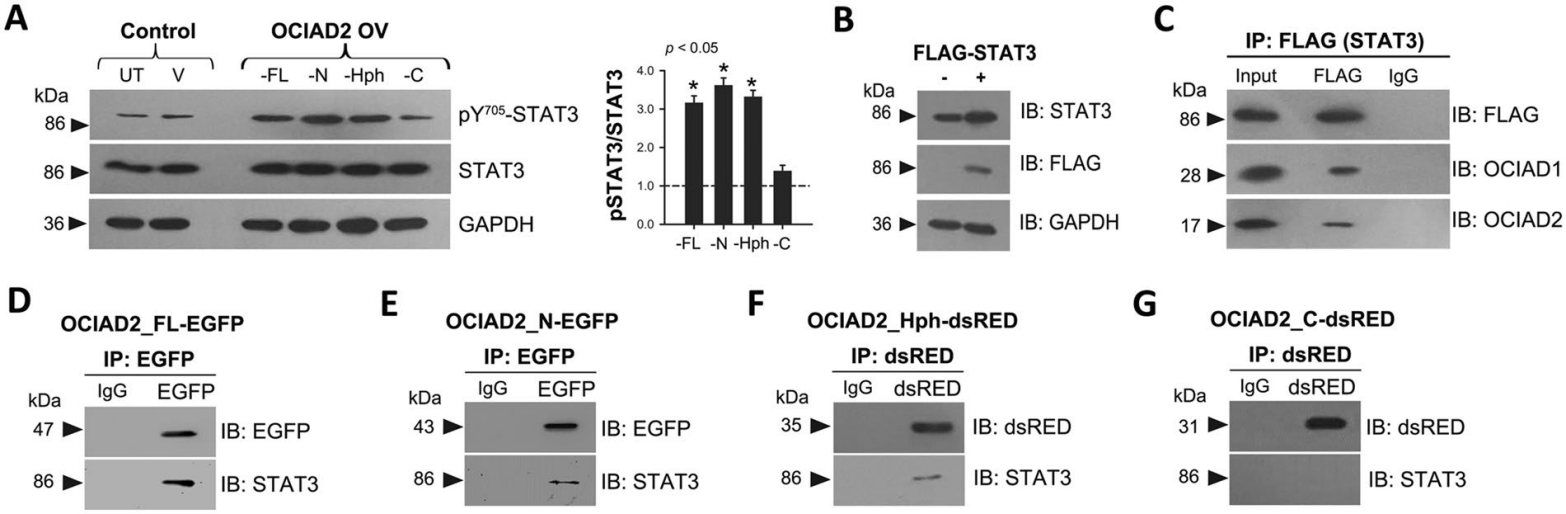
The double helical motif of OCIAD2 interacts with STAT3 and promotes its activation. (**A**) Western Blotting for detection of STAT3 and pSTAT3 (Y705) levels in HEK293 cells transfected with various OCIAD2 constructs as indicated. UT: untransfected, V: empty vector transfected; FL: OCIAD2_FL-EGFP; N: OCIAD2_N-EGFP; Hph: OCIAD2_Hph-dsRed and C: OCIAD2_C-dsRed. Graph represents pSTAT3/STAT3 ratio obtained upon overexpression of various OCIAD2 constructs relative to control (shown as a dotted line). n = 4 (**B**) Validation of transfection of FLAG-STAT3 construct into HEK293 cells by Western Blotting. (**C**) FLAG-STAT3 transfected HEK293 cell lysate subjected to immunoprecipitation with anti-FLAG antibody and assessed for interaction with OCIAD1 and OCIAD2. (**D**–**G**) HEK293 cell lysates expressing various OCIAD2 constructs (FL, N, Hph and C) were subjected to immunoprecipitation (IP) followed by immunoblotting (IB) to probe for interaction with STAT3. Protein size markers (in kDa) are indicated to the left. Data for STAT3/pSTAT3 Western Blotting are representative of three independent experiments and *p < 0.05 was used to indicate a statistically significant difference. At least two independent experiments with two technical replicates each were performed and similar results were obtained. Full-length blots are presented in Supplementary Figure S6.

Given that the OCIA domain of OCIAD2 is sufficient for proper localization and OCIAD1/Asrij interaction, we next tested whether it could provide function. As Asrij activates STAT3^9^ and OCIAD2 is implicated in several cancers where STAT3 is active, we checked for any functional homology between the OCIAD proteins by testing the ability of different regions of OCIAD2 to bring about STAT3 activation. HEK293 cells overexpressing OCIAD2_FL-EGFP showed increased levels of activated STAT3 without affecting total STAT3 levels (Fig. 6A). To define the domain of OCIAD2 essential for promoting STAT3 phosphorylation, we transfected HEK293 cells with various OCIAD2 constructs and validated expression (see Methods and Fig. S5A,B). OCIAD2_N-EGFP or OCIAD2_Hph-dsRED expressing cells had significantly increased phosphorylated STAT3 (Y705) levels as compared to the controls, unlike OCIAD2_C-dsRED, which showed no significant change (Fig. 6A). These results reveal a previously undescribed role for the helical motif of OCIAD2 (41–117 aa) in promoting phosphorylation of STAT3.

### The double helical motif of OCIAD2 interacts with STAT3

The OCIA domain of Asrij is the functional domain that binds STAT3 in mouse embryonic stem cells and to the trafficking protein ADP Ribosylation Factor 1 (ARF1) in *Drosophila*^12,28^. To understand the functional interplay between the OCIA domain proteins, we checked whether OCIAD2 and Asrij had common interacting partners and mediated similar cellular functions. From HEK293 cells expressing FLAG-tagged STAT3 construct (Fig. 6B), we found that OCIAD2 co-immunoprecipitates with STAT3 (Fig. 6C). Furthermore, by immuno-pulldown from cells expressing OCIAD2_FL-EGFP or OCIAD2_N-EGFP or OCIAD2_Hph-dsRed or OCIAD2_C-dsRed we also found that the helical motif of OCIAD2 (aa 41–117) is necessary and sufficient for mediating interaction with STAT3 (Fig. 6D–G). The interaction of OCIAD2 with STAT3 could be direct or indirect. Given that Asrij and OCIAD2 interact, it is likely that Asrij acts as a scaffolding partner, as suggested earlier^9^ and helps in mediating OCIAD2-STAT3 interaction or vice-versa. Our findings suggest that both Asrij and OCIAD2 are functional homologs for STAT3 activation and signaling.

### OCIAD2 depletion leads to reduced STAT3 activation and cell migration

**Figure 7:**
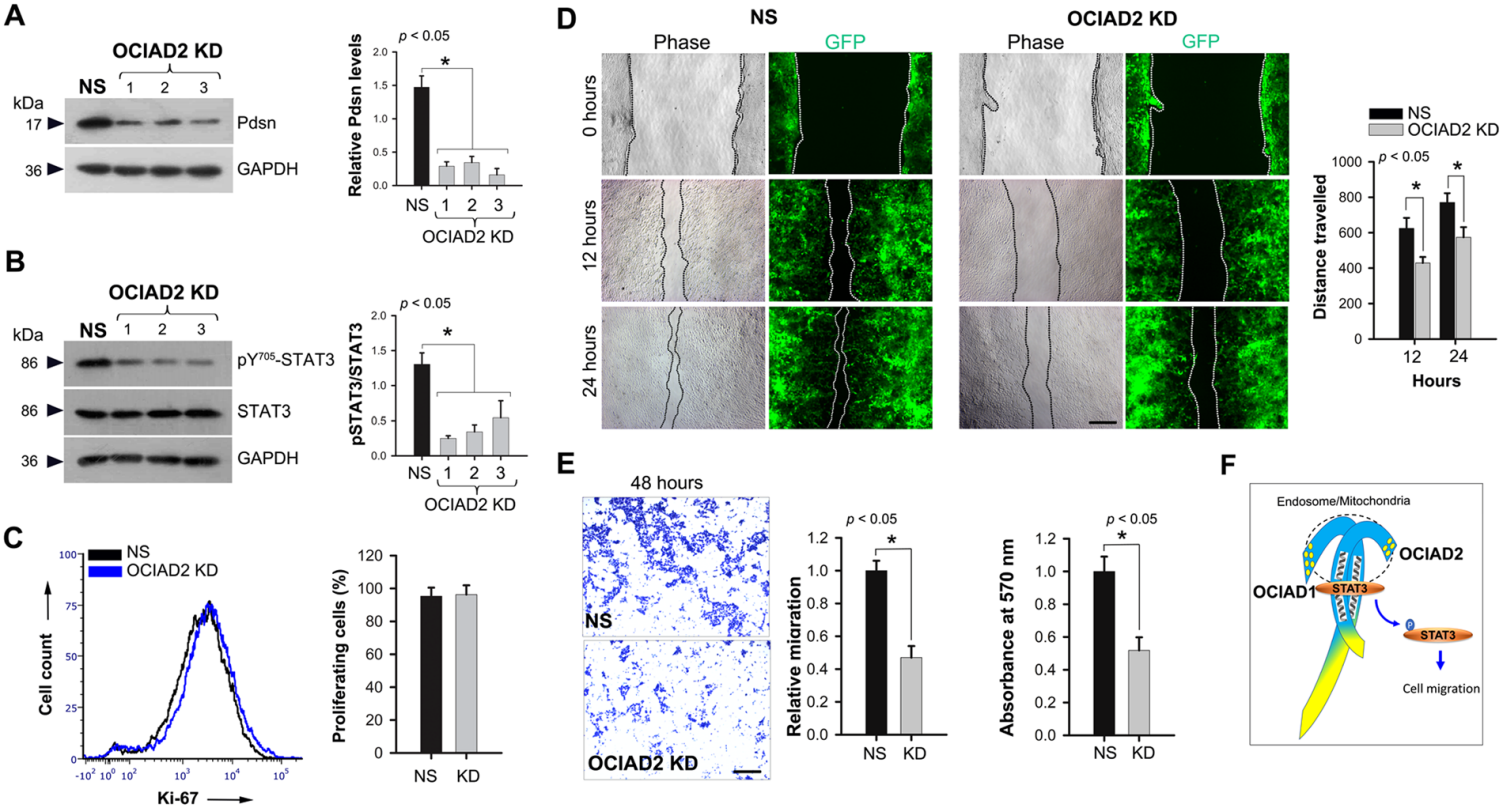
Knockdown of OCIAD2 retards migration of HEK293 cells. (**A**) Western Blotting confirming *ociad2* knockdown (KD) in OCIAD2_ shRNA (1, 2 and 3) transfected HEK293 cells as compared to non-silencing (NS) shRNA. Graph shows relative OCIAD2 levels in NS and KD. n = 4 (**B**) Western Blotting for detection of STAT3 and pSTAT3 (Y705) levels in NS and KD. Data are representative of three independent experiments and graph shows relative STAT3/pSTAT3 ratio upon *ociad2* KD. Protein size markers (in kDa) are indicated to the left. Full-length blots are presented in Supplementary Figure S6. n = 4 (**C**) Flow cytometry analysis of Ki-67^+^ population in *ociad2* KD HEK293 cells. Graph shows results obtained from four independent experiments (mean ± SEM). (**D**) Reduced migration of *ociad2* KD cells observed in wound healing assays. Monolayers of control and *ociad2* knockdown HEK293 cells were wounded and the degree of recovery was measured at 0, 12 and 24 hours post-wounding. Representative phase and fluorescence images, 4× magnification. Measurement and estimation of wound recovery was based on the initial wound size. Graph shows quantification of results obtained from three independent experiments (mean ± SEM) with at least 5 fields analyzed per sample per experiment. (**E**) Representative images (4× magnification) showing reduced migration of *ociad2* KD HEK293 cells after 48 hours in a trans-well migration assays. Graphs represent quantification of cell migration (absorbance at 570 nm) obtained from two independent trans-well experiments performed in two technical replicates (mean ± SEM). *p < 0.05 was used to indicate a statistically significant difference. (**F**) Proposed model depicting the location of OCIAD1 and OCIAD2 and their interaction via the double helical motif (wavy grey lines). Blue shading: conserved region; Yellow: non-conserved regions. STAT3 interaction with OCIAD1 or OCIAD2 (individually or together) leads to STAT3 activation essential for cell migration.

Since increased OCIAD2 levels result in enhanced STAT3 activation, we tested the effect of OCIAD2 depletion on STAT3 activation and cellular function. ShRNA-mediated knockdown (KD) of *ociad2* transcripts in HEK293 cells with three different shRNA constructs (OCIAD2_ shRNA 1, 2 and 3) or non-silencing shRNA control (NS) (see Methods) gave over 75% reduction in protein levels (Fig. 7A). OCIAD2 depletion caused a significant reduction in STAT3 activation (Y705) as compared to NS control (Fig. 7B).

An important role of JAK/STAT signaling in development, immunity and cancer is to promote cell proliferation and migration. Ki-67 staining of proliferating cultures showed that OCIAD2 knockdown did not affect cell proliferation (Fig. 7C). However, OCIAD2 knockdown reduced cell migration in HEK293 cells in a wound healing assay as seen at 12 hours and 24 hours post-scratch (Fig. 7D). Additionally, in a trans-well assay, migration by OCIAD2 depleted cells was much reduced (40%) compared to non-silencing control (Fig. 7E). However, overexpression of OCIAD2 full-length or various fragments showed no significant change in migration or proliferation of HEK293 cells (Fig. S5C–E). Taken together, these data suggest a differential function for OCIAD2, in aiding the role of JAK/STAT signaling in cell migration but not cell proliferation.

## Discussion

In spite of important implications in normal development and pathological conditions^4–7^, the structure or *in vivo* vertebrate function of OCIA domain proteins is unreported. In the present study, we provide the evolutionary history of the OCIAD gene family and report the gene family repertoire for the first time in several vertebrate and non-vertebrate species. Our detailed *in silico* analysis shows that *ociad2* is a genomic neighbor of *ociad1* in most vertebrates. Mapping the chromosomal arrangement of *ociad1* and *ociad2* with respect to the conserved flanking genes *fryl* and *cwh43* showed that the gene synteny is strongly conserved in mammals, primates, birds and reptiles. Although *cwh43* is lacking in some species and *ociad1* and *ociad2* are located on different chromosomes in some teleost fish (*Ictalurus punctatus*, *Oreochromis niloticus*, *Oryzias latipes*), the gene synteny is conserved with respect to the next flanking gene *dcun1d4*. Apart from the conserved genomic arrangement and gene synteny of *ociad1* and *ociad2*, we found that the intron-exon structure of *ociad1* and *ociad2* is also conserved between mammals and zebrafish. Comparison of their intron-exon structure shows that there are extra exons towards the 3′ of *ociad1* as compared to *ociad2* in both mammals and zebrafish, suggesting that these structural differences were already present in the last common ancestor of these species. Taken together, these results and our phylogenetic analysis strongly indicate that the vertebrate *ociad1* and *ociad2* genes are co-orthologs of the invertebrate *ociad*. The two vertebrate genes likely arose as a consequence of tandem duplication of an ancestral *ociad* gene in the last common ancestor of jawed vertebrates around 435–500 mya.

Interestingly, our *in silico* analysis also showed an extra *ociad1-like* gene in some teleost fish such as zebrafish (*Danio rerio*) and Atlantic herring (*Clupea harengus*), resulting in three copies of *ociad* in these species, unlike other members such as catfish (*Ictalurus punctatus*), tilapia (*Oreochromis niloticus*) and medaka (*Oryzias latipes*), which have only two copies of *ociad*, on different chromosomes (Fig. 1). Apart from *ociad1a* and *ociad2a* on chromosome 20, zebrafish has an additional gene *ociad1b* on chromosome 1. A similar arrangement of the *ociad1a*, *ociad2a* and *ociad1b* genes is found in Atlantic herring. In zebrafish, chromosome 20 and chromosome 1 are paralogous chromosomes that arose from whole genome duplication (WGD). Hence we speculate that while *ociad1b and ociad2b* may have originated as a result of WGD during the evolution of teleost fish^29^, subsequently, *ociad2b* as well as its flanking genes *fryl* and *cwh43* may have been lost. This is further supported by the presence of additional duplicated genes such as *fip1l1a* (chromosome 20, near *ociad1a*) and *fip1l1b* (chromosome 1, near *ociad1b*). Further, this also suggests that catfish, tilapia and medaka could have secondarily lost one of their *ociad1* copies, the one located next to *ociad2* and *fryl* (on chromosomes 25, LG23 and 4, respectively), and that their remaining *ociad1* genes (on chromosomes 29, LG6 and 1, respectively) are not orthologs of zebrafish *ociad1* from chromosome 20, but their WGD paralogs. Thus, although vertebrate *ociad1* and *ociad2* arose by tandem duplication from an ancestral *ociad* gene, WGD seems to be the most likely explanation for the presence of *ociad* genes on different chromosomes in teleosts.

We also found that *ociad2* was absent in some species (*Gallus gallus*, *Rhincodon typus*, *Lepisosteus oculatus*). This could be due to complete or partial loss of some regions of *ociad2* after duplication during evolution of these species due to random events (such as drift) or redundancies in OCIAD2 function. The perceived absence may also be due to gaps in genome assembly (as in the case of *Rhincodon typus*).

Gene duplication events have been recognized as key players in creating chromosomal variation and evolutionary change^30,31^. Many novel proteins arise as a result of gene duplication^32^ and duplicated genes thus formed, often generate functional redundancies^33^. Moreover, duplicated genes can have various possible fates^34^- for example, both the parental and duplicated gene may have identical sequence and function, perhaps leading to a higher level of gene expression, or one copy may accumulate point mutations resulting in a pseudogene^35^. Additionally, mutations acquired as a result of gene duplication may also lead to sub-functionalization or neo-functionalization^36^.

Interestingly, a pseudogene, OCIAD2P1 is annotated for human *OCIAD2* in GenBank (*OCIA domain containing 2 pseudogene 1*; Gene ID: 100287085) and present on chromosome 8 (8q21.13) and is 87% identical to human *ociad2* transcript variants 1, 2 and 3, suggesting that it is a processed pseudogene or a retrocopy. This seems to have arisen by mRNA-mediated gene duplication of *ociad2* and is specific only to humans as we do not find any annotated *ociad2* pseudogene in other species. It is possible that *ociad2* is undergoing a drift or a positive selection in humans and this pseudogene is likely to have non-coding RNA regulatory roles.

The chromosomal proximity along with largely overlapping developmental expression patterns and sub-cellular localization of OCIAD2 with Asrij suggest that the gene duplication could provide functional redundancy between these two genes in some tissues and temporal windows. The presence of multiple alternatively spliced transcripts suggests complex regulation of this gene. Comparison of the intron-exon organization of the longest transcript of the *ociad* genes in *Danio rerio*, *Mus musculus* and *Homo sapiens* (Fig. 3A) showed that the OCIA domain maps to the same exons and all variations are outside the domain region. Interestingly, few splice variants were truncated within the domain raising the possibility of non-functional isoforms. However, no additional protein isoforms were detected suggesting transcriptional or post-transcriptional regulation of *ociad2*, which merits further investigation.

Regulatory region mutations could generate differences in spatial or temporal regulation of the duplicated genes^37,38^. The reverse orientation of *asrij* and *ociad2* with respect to each other, makes it very likely that they may have non-overlapping upstream regulators, though they could be regulated by common enhancers. Indeed, bioinformatics analysis suggests that they have very few transcription factor binding sites in common (see Methods). The expression pattern of OCIAD2 varies across different mouse tissues and carcinomas. It is noteworthy that while OCIAD2 expression is higher in kidney, liver and brain relative to other tissues sampled, carcinomas of the liver and brain have significantly reduced OCIAD2 expression. On the other hand, OCIAD2 expression is low in the ovary whereas ovarian carcinomas have increased expression of OCIAD2. Thus, tissue-specific differences in OCIAD2 expression levels co-relate inversely with its status in carcinomas. However, our analysis indicates that while change in OCIAD2 level affects STAT3 activation, it does not affect proliferation. Hence, OCIAD2 may aid in selecting different outcomes of JAK/STAT signaling. Also, additional factors or events are likely involved in selecting downstream effects of JAK/STAT signalling. It is noteworthy that in most tissues and cell lines tested, Asrij expression levels are higher than those of *ociad2*. The significance of this is unclear but could impact the outcome of signaling mediated by the two genes.

Unlike earlier studies, wherein carcinoma cells (HeLa) were transfected with GFP/RFP-tagged constructs of OCIAD2 in combination with reporter-tagged intracellular markers^19^, our approach avoids bias or mis-localization and informs about the subcellular localization of OCIAD2. Further, our comparative analysis with OCIAD1 provides insight into the conserved OCIA domain family proteins, their interaction, localization and function (Fig. 7F). The sub-cellular location of OCIAD2 is primarily in mitochondria, whereas Asrij localizes to multiple endocytic vesicles and mitochondria. Further, the localization of OCIAD2 N-terminal fragment is similar to that of endogenous OCIAD2 (Fig. 4B), reminiscent of Asrij N-terminal^9^. Insects have only one OCIA domain protein (Asrij), whereas vertebrates express two (OCIAD1/Asrij and OCIAD2), suggesting that these proteins have important functions that need to be enhanced or additionally regulated in vertebrates by an additional gene. OCIAD2 is primarily composed of the OCIA domain, thus highlighting the importance of this domain in mediating protein function. Our analysis additionally narrows down to a 77-aa stretch of the domain, showing that the double helical motif is sufficient for proper localization.

The C-terminal fragment of OCIAD2 is not sufficient for localizing the protein correctly and does not bind to Asrij or STAT3. OCIAD2 sequence between amino acids 120–154, the non-domain region, is only 14.7% identical to the corresponding region in Asrij. This suggests that the non-domain regions of the two proteins may differ greatly in their functional relevance. In the case of both Asrij and OCIAD2, the non-OCIA domain region has no predicted structure. Intrinsically unstructured proteins are known to adopt a conformation depending on their interactors and can also act as scaffolds. It is possible that such interactions help the conserved OCIA domain achieve stability. This opens up new and very interesting possibilities for regulation of OCIAD family proteins in development and disease that can be tested.

Recent studies show that endosomes and mitochondria interact directly^39^ and that endosome-mitochondrial trafficking plays an important role in regulation of metabolite homeostasis^40^. The subcellular localization and interaction of the OCIA-domain containing proteins in both these compartments leads us to speculate that they could be involved in trafficking of endosomal cargo and cross talk between endosomes and mitochondria. Recent high throughput studies indicate that both OCIAD1 and OCIAD2 localize to mitochondria^27,41^. Given that there is a large overlap in the expression patterns of OCIAD proteins and that the OCIA domain acts as a dimerization domain, it will be interesting to test whether OCIAD2 has a supportive or inhibitory role, possibly dominant negative, on OCIAD1 function. Further, since both proteins are implicated in neurodegenerative diseases such as Alzheimer’s and Parkinson’s it raises the possibility that their aberrant dimerization or heterodimerization may contribute to aberrant endosomal functions or plaque formation, which needs to be explored. The OCIA domain of Asrij binds STAT3 and promotes its activation. We show that OCIAD2 too interacts with STAT3 and OCIAD2 levels correlate directly with activated STAT3. Further, the double helical region of OCIAD2 (aa 41–117) is necessary and sufficient for promoting STAT3 activation.

Earlier, we have shown that Asrij and STAT3 can reside on the same endosomal compartment and that Asrij promotes endosomal activation of STAT3^9^. Given that OCIAD2 is also present on mitochondria and a subset of Rab5-positive early endosomes and that it promotes STAT3 activation, it is likely to have a role in mediating activation of STAT3. Apart from endosomal activation, the mitochondrial activation of STAT3 has also been reported^42^. It will be interesting to test whether OCIA domain proteins could have roles in regulating STAT3 activation in mitochondria too.

Members of a protein family often exhibit a high level of functional redundancy and compensation. Similar developmental profiles and expression patterns of OCIAD1/Asrij and OCIAD2 suggest their regulation may be spatio-temporally coordinated. We propose that human OCIA domain proteins may similarly co-express and possibly have inter-linked or redundant functions. Further studies will help elucidate the role of OCIAD2 in vesicular trafficking, development and maintenance of stemness, which will also be applicable to understanding human OCIAD2 in disease.

## Materials and Methods

### Phylogenetic analyses of OCIAD family proteins

Protein sequences selected for performing phylogenetic analysis have been listed in Supplementary Table S1 and were included based on the phylogenetic position of different species. Before proceeding with the phylogenetic analysis, the multispecies protein sequence alignment was assessed for unusual gaps, insertions or missing data. Isoforms of proteins have been ignored for analyses, unless in cases where full-length sequences were not available. In those cases, the longest isoform was used under the assumption that it will most fully represent the full-length sequence. A total of 106 amino acid sequences of invertebrate OCIAD and vertebrate OCIAD1/Asrij and OCIAD2 from 58 different species (see Supplementary Table S1) were retrieved from NCBI (https://www.ncbi.nlm.nih.gov/), aligned using the UPGMA (Unweighted Pair Group Method with Arithmetic Mean) clustering algorithm in MUSCLE^21,43^ implemented using MEGA7^44^. These sequences were then used to identify suitable models of amino acid substitutions using an automated maximum likelihood Neighbor-Joining tree method (model selection analysis) available in MEGA7^44^. 56 such models were tested, and the model with the least Bayesian Information Criterion (BIC) was selected as the one explaining the substitution pattern the best (see Supplementary Table S2). The pattern of amino acid substitutions in our sequences seemed to be explained best by a JTT + G model (+*G*, parameter = 0.7508). The sequences were then used to construct a phylogenetic tree, to understand the evolutionary relationships between OCIAD family members. The OCIAD family tree was constructed using the Maximum Likelihood method based on the JTT matrix-based model. The phylogenies were tested using bootstrap with 100 replicates. We used the ‘find gene duplication’ wizard in MEGA7 to identify locations in the tree that may have undergone gene duplication events to further understand the relationship between OCIAD, OCIAD1/Asrij and OCIAD2. MEGA7 requires the gene tree to be rooted for the gene duplication analysis, and we used the OCIAD *Stylophora pistillata* branch as the root of the tree to proceed with the analysis.

### Estimating rates of amino acid change across lineages

For a comparative analysis of the rate of amino acid changes between the two OCIAD family members, the number of sites that are identical between the OCIAD1 and OCIAD2 human sequences with that of their counterparts in birds, reptiles, amphibians, fish and other mammals were retrieved using EMBOSS Needle (https://www.ebi.ac.uk/Tools/psa/emboss_needle/). The difference between number of identical sites and the length of the protein sequence in the respective species was calculated and scaled by the entire length of the protein and multiplied by a factor of 10 to account for amino acid change per 10 residues. Human OCIAD1 and OCIAD2 sequences are considered the most recent ones, and therefore the evolutionary time reference of human OCIAD proteins has been set to 0 million years ago (mya). The average changes for different classes were calculated, and radiations of OCIAD1/Asrij and OCIAD2 were plotted against mya beginning from the earliest known record of each class using Histone-3 as a reference owing to its highly-conserved nature^45^. Linear regressions were performed using SigmaPlot11 to estimate best fits and thereby, the linear rates of amino acid change in the protein sequences of interest.

### Animals, Tissues and Cell lines

All animal experimental protocols were approved by Institutional Animal Ethics Committee (IAEC) of JNCASR (Project number MSI005). All animals were maintained, and experiments performed according to the guidelines and regulations of the IAEC, JNCASR. Cell cultures were maintained as described earlier^46^.

### RT-PCR

Total RNA was extracted from mouse tissues using TRIzol reagent (Thermo Fisher Scientific, USA) following the manufacturer’s instructions. 2 µg of DNase-treated RNA was converted to cDNA by reverse transcription using Superscript II (Invitrogen, USA) and then amplified using combinations of the following forward (F) and reverse (R) primers: mOCIAD2_F1 5′AGGGAAACAGTTGGAGTCGC3′; mOCIAD2_F2 5′ACGCGTCGACGCCATGGCTTCAGTGTCC3′; mOCIAD2_F3 5′AGGAGTCGACGCCATGGTTATCAGAGAGTG3′; mOCIAD2_F4 5′TCACCCAGGGACTCGTCCAC3′; mOCIAD2_R1 5′GAG AGGATCCCTCGAGAG CAGATGGTTGTG3′; mOCIAD2_R2 5′AGCCGGATCCCTCGAGCTGATCTTCAAAG3′ and mOCIAD2_R3 5′CCAAATCTTGGATTAGCGGC3′. All reactions were performed on three independent biological samples taking duplicates per reaction.

### *In silico* analysis

For identification of *ociad2* in whale shark (*Rhincodon typus*), we performed a tblastn (https://blast.ncbi.nlm.nih.gov/Blast.cgi?PROGRAM=tblastn&PAGE_TYPE=BlastSearch&LINK_LOC=blasthome) search against the genome of whale shark using elephant shark (*Callorhinchus milii*) OCIAD2 (Accession ID: XP_007890924.1) as the query sequence. For detecting homology of OCIAD proteins to any other known transactivation domains, protein sequences were submitted to an online program Blast (https://blast.ncbi.nlm.nih.gov/Blast.cgi). For analysis of *ociad2* transcript levels in various human tissues and cell lines, we used RNAseq datasets available on VastDB (http://vastdb.crg.eu/wiki/Main_Page). The gene names “ociad1” and “ociad2” for human were provided as the search terms and datasets corresponding to gene IDs ENSG00000109180 and ENSG00000145247 were downloaded and CRPKM (control reads per kb of interrogated region per total million mapped reads) values were analyzed to compare the transcript-expression levels of *ociad1* and *ociad2*. For predicting structure of zebrafish, mouse, and human OCIAD1 and OCIAD2 proteins, various structure prediction programs such as RaptorX (http://raptorx.uchicago.edu/) and Phyre^2^ (http://www.sbg.bio.ic.ac.uk/phyre2/html/page.cgi?id=index) were used wherein the amino acid sequences of these proteins corresponding to Uniprot IDs Q6NYD7, Q9CRD0, Q9NX40, Q5RHX2, Q9D8W7 and Q56VL3 were fed as the input. Hydrophobicity plots for mouse OCIAD1 and OCIAD2 sequences were predicted using TmPred (http://www.ch.embnet.org/software/TMPRED_form.html) web server with the default settings. Analysis of transcription factor binding sites in OCIAD1 and OCIAD2 was done using tools such as Genomatix software suite v3.8, UCSC genome browser (https://genome.ucsc.edu/) and CODEX (codex.stemcells.cam.ac.uk/).

### Generation of tagged expression constructs

OCIAD2 fusion constructs with EGFP and dsRed were generated by introducing various OCIAD2 fragments upstream of EGFP and dsRed sequences into the vectors pEGFP-N3 and pdsRED2-N1, respectively (Clontech) between SalI and BamHI restriction sites. OCIAD2_FL-EGFP contained full- length protein (1–154 aa), OCIAD2_N-EGFP the OCIA domain (1–117 aa), OCIAD2_Hph-dsRED (41–117 aa) and OCIAD2_C-dsRed the C-terminal of OCIAD2 (113–154 aa).

### Cell culture, transfection and immunostaining

2.5 × 10^4^ HEK293 cells maintained in DMEM media containing 10% FBS were transfected after 36 hours with 1.0 μg of OCIAD2 reporter constructs (OCIAD2_FL-EGFP, OCIAD2_N-EGFP, OCIAD2_Hph-dsRED and OCIAD2_C-dsRED). After 48 or 72 hours, the transfected cultures were washed with PBS, fixed with 2% paraformaldehyde and processed for immunostaining using standard procedures as described previously^9^.

For *ociad2* knockdown three independent shRNAs targeting human *ociad2* (V3LHS_391531 (OCIAD2_ shRNA 1), V3LHS_391532 (OCIAD2_ shRNA 2), V3LHS_391533 (OCIAD2_ shRNA 3)) and control GIPZ shRNA V2LHS_15681 (non-silencing) bearing TurboGFP expressed from pGIPZ-puromycin lentiviral vectors were obtained from Dharmacon, GE (USA). HEK293 cells were transfected as above. The following day, the medium was replaced and cells were incubated at 37 °C for 36–48 hours in the presence of 1 µg/mL puromycin to select GFP-positive cells. This procedure was repeated for selection until 90–95% cells were GFP-positive. These cells were further expanded in culture in the presence of puromycin, for use in all experiments.

For immunostaining, primary antibodies used were against: Rab5 (Cat. No. 3547), Rab7 (Cat. No. 9367) and Rab11 (Cat. No. 5589) (all from Cell Science Technologies, USA); CoxIV (Cat. No. ab33985) and OCIAD1 (Cat. No. ab91574) (Abcam, USA); GM130 (Cat. No. 610822, BD Biosciences, USA) and OCIAD2 (Cat. No. HPA041090, SIGMA, USA). Secondary antibodies were coupled to Alexa-Fluor 488 or Alexa-Fluor 568 (Molecular Probes). Imaging was done using Carl Zeiss LSM510Meta and LSM880 microscopes. Images were processed in LSM software and adjusted uniformly for brightness/contrast using Adobe Photoshop CS3.

### Co-localization index calculation

Co-localization of OCIAD2 with various markers was performed using the ZEN image processing software (ZEN, Carl Zeiss Inc). Using the “Overlay” tool, a ROI was drawn around the cell of interest. Single optical slices from Z-stack images of HEK293 cells captured using confocal microscopy were thresholded, followed by superimposition of the green and red channels to measure the co-localization percentage. Statistical data for the co-localization levels, co-localization coefficients for green and red channels, overlap coefficient (*R*), Pearson’s correlation coefficient (*Rr*) was generated using the “Coloc” option in the software. The number of co-localized pixels divided by the total number of green and red pixels was presented as the co-localization percentage.

### Cell/tissue lysis and Western Blotting

Western Blotting of HEK293 cells and mouse tissues was done as described previously^9^. Antibodies used for Western Blotting analysis were directed against OCIAD1, OCIAD2 as above, FLAG (Cat. No. F7425, SIGMA, USA) EGFP (Cat. No. A11122, Invitrogen, USA), dsRED (632496, Clontech, USA), STAT3 (9139 S, Cell Science Technologies, USA), phospho-STAT3 (9154 S, Cell Signalling Technologies, USA). Lysates were normalized with respect to loading control glyceraldehyde 3-phosphate dehydrogenase (GAPDH) (Cat. No. G9545, SIGMA, USA). Secondary antibodies used were anti-rabbit or anti-mouse HRP-conjugated.

### Immunoprecipitation

For immunoprecipitation studies involving OCIAD1 and OCIAD2, Protein G sepharose beads (SIGMA) coated with desired antibodies were incubated with HEK293 cell lysates for 4 hours, clarified by centrifugation and extensively washed. Equal volumes of sample were loaded and resolved by SDS polyacrylamide gel and processed for Western blot analysis. For testing STAT3 and OCIAD2 interaction, cell lysates of HEK293 transfected with FLAG-STAT3 construct^9^ were incubated with anti-FLAG antibody and similarly taken for immunoprecipitation. At least two independent immunoprecipitation experiments with two technical replicates were performed and similar results were obtained.

### Densitometry analysis

To quantitatively determine the extent of *ociad2* knockdown or fold change in STAT3 and phospho-STAT3 levels (pSTAT3/STAT3 ratio) in respective cell lysates, we measured the density and pixel counts for each band using ImageJ 1.48 v software and normalized the values to GAPDH for plotting.

### Wound healing assay

HEK293 cells were seeded at 25,000 per well to a 4-well dish (Nunc, USA) and grown to form a monolayer. After serum starvation (2% FBS + DMEM + L-glutamine) for 12 hours, the monolayer was scratched (wounded) with a sterile 200 μl pipette tip, debris was removed by washing the cells with 1 mL of growth medium (10% FBS + DMEM + L-glutamine) per well and the medium was replaced with 300 µL growth medium per well. Phase and fluorescence images were acquired at 0, 12 and 24 hours post-wounding intervals using an IX-70 inverted microscope (Olympus, Tokyo, Japan) equipped with a digital camera. The difference between the initial and final wounded areas were calculated using the ImageJ 1.48 v software and the results obtained were used to estimate the degree of recovery.

### Trans-well migration assay

*In vitro* migration and invasion assay was performed in 24-well plates using 8.0-µm pore polycarbonate trans-well inserts (BD Corning). 5 × 10^4^ cells were suspended in serum-free DMEM and overlaid in the upper chamber of each trans-well. DMEM medium containing 10% FBS was added to the lower chamber as a chemoattractant. The inserts were then incubated at 37 °C in a humidified atmosphere containing 5% CO_2_. After 36 and 48 hours of incubation, cells that failed to penetrate the pores of the filter were removed by cotton swabs and the migrated cells were fixed and stained with 0.1% crystal violet (C0775, SIGMA, USA). Images of crystal violet stained cells were acquired with the help of an IX-70 inverted microscope (Olympus, Tokyo, Japan) equipped with a digital camera. Quantification of migrated cells from at least 5 different microscopic fields per sample was done using ImageJ 1.48 v software. The migratory activity was calculated as the average number of migrated cells in each assay from three independent experiments. Further, migratory activity was also assayed by dissolving cells that invaded the filter in methanol and measuring absorbance at 570 nm using a spectrophotometer.

### Ki-67 cell proliferation assay

Control or *ociad2* knockdown or overexpressing HEK293 cells were fixed, permeabilized and stained with Ki67-FITC conjugated antibody (Cat. No. 556026, BD Biosciences, USA) or Ki67-PE conjugated antibody (Cat. No. 556027, BD Biosciences, USA) depending on the EGFP/dsRED tagged constructs used for transfection. Proliferation was analyzed by flow cytometry (FACSAria II, BD Biosciences, USA) and the results were interpreted using software FCS Express 6.

### Statistical analysis

All experiments were performed on at least three independent biological samples with two technical replicates. Single factor ANOVA or Student’s *t*-test were used to make relevant comparisons depending on the experimental design using Analysis ToolPak in Microsoft Excel 2007. Means ± SEM were plotted as graphs using SigmaPlot11.0. All results were considered significant at *p* < 0.05.

### Data availability

All data generated or analyzed during this study are included in this published article (and its Supplementary Information files). All manuscript data are available in the main or supplementary files uploaded.

## Electronic supplementary material

supplementary text and figures

Dataset 1

Dataset 2
