# Supplementary material for: A double helical motif in OCIAD2 is essential for its localization, interactions and STAT3 activation
Source: Sci Rep. 2018 May 9;8:7362. doi: 10.1038/s41598-018-25667-3 (PMC5943604; doi:10.1038/s41598-018-25667-3)
Supplement: Supplementary file 1 — supplementary text and figures [file 41598_2018_25667_MOESM1_ESM.pdf]

A double helical motif in OCIAD2 is essential for its localization, interactions and STAT3 activation.

Saloni Sinha[1], Anudeep Venkata B[1] and Maneesha S. Inamdar[1,2*]

[1]Jawaharlal Nehru Centre for Advanced Scientific Research, Jakkur, Bangalore 560064, India; [2]Institute for Stem Cell Biology and Regenerative Medicine, GKVK, Bellary Road, Bangalore 560065, India.

Inventory of Supplementary Information

1. Supplementary information
    a. Supplementary Figure S1
    b. Supplementary Figure S2
    c. Supplementary Figure S3
    d. Supplementary Figure S4
    e. Supplementary Figure S5
    f. Supplementary Figure S6
2. Supplementary Table S1
3. Supplementary Table S2
4. Revised main text with tracked changes

Supplementary Information

Supplementary Figure legends

A

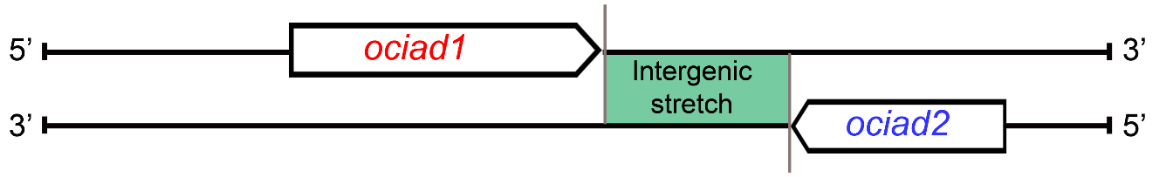

B

Species	*ociad1*		Intergenic stretch (nt)	*ociad2*	
	Start (nt)	Stop (nt)		Stop (nt)	Start (nt)
Homo sapiens	48830924	48861817	23563	48885380	48906828
Pan troglodytes	78515364	78456232	24010	78432222	78410576
Felis catus	166630569	166657489	34818	166622671	166607115
Canis lupus	44608474	44634665	18816	44653481	44639044
Mus musculus	73292769	73314077	24870	73338947	73322198
Rattus norvegicus	37458023	37475485	26060	37449425	37432999
Taenopygia guttata	44782196	44797852	22554	44775298	44763625
Xenopus tropicalis	29962953	29995438	19559	30014997	30007294
Danio rerio	23245620	23253570	14737	23238833	23244193

Fig. S1. Comparative analysis of nucleotide positions of *ociad1* and *ociad2* across representative species. (A) Schematic representing the transcriptional direction of *ociad1* and *ociad2*. **(B)** Table showing the nucleotide (nt) positions of *ociad1* and *ociad2* and the intergenic stretch between them in species where the genes are neighboring.

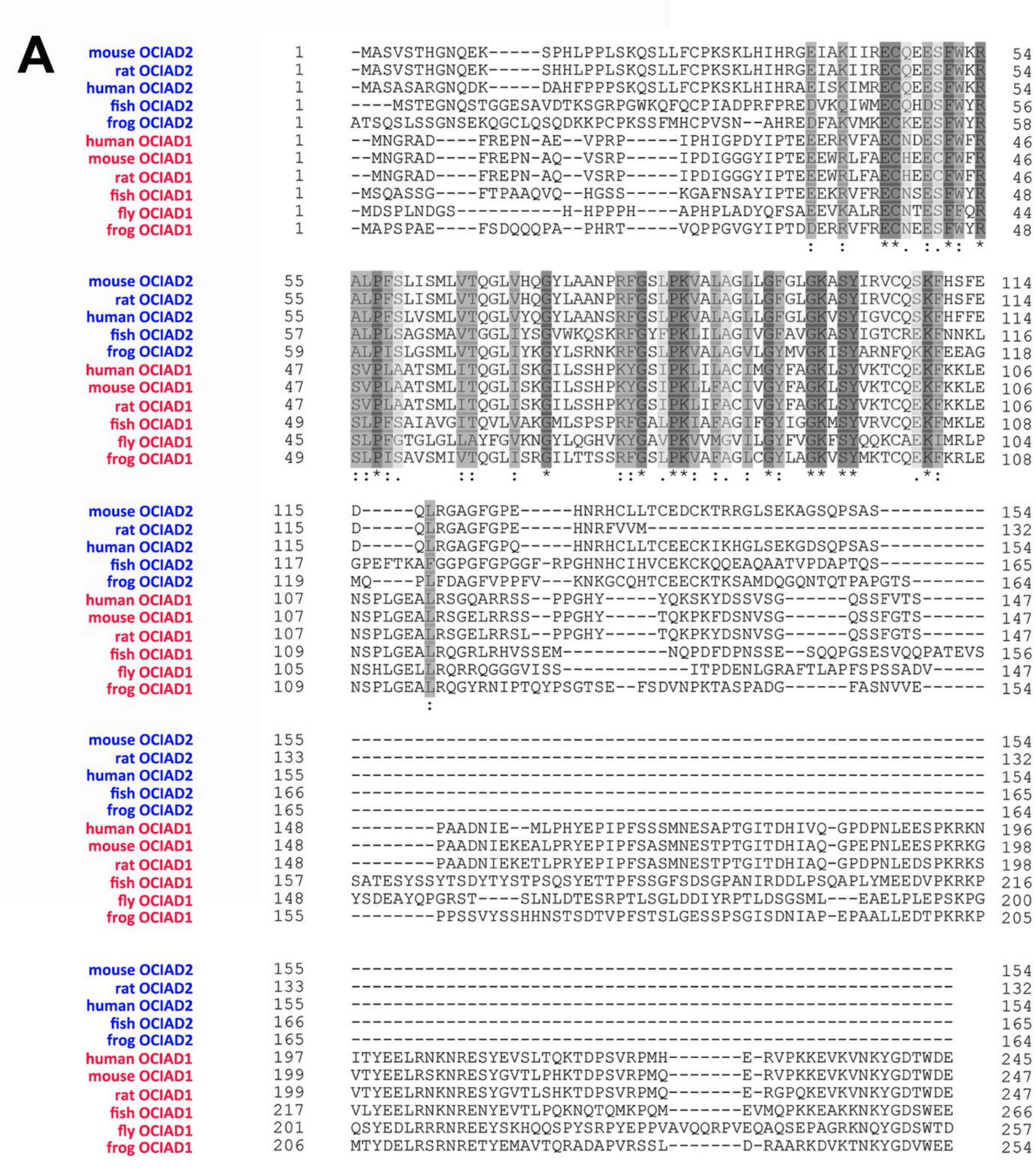

Fig. S2. Protein sequence alignment of OCIAD1 and OCIAD2 for different species. (A) Multiple sequence alignment of OCIAD1 and OCIAD2 protein sequences from various organisms

(using Clustal-Omega) showing sequence conservation in the N-terminal regions as opposed to the C-terminal regions.

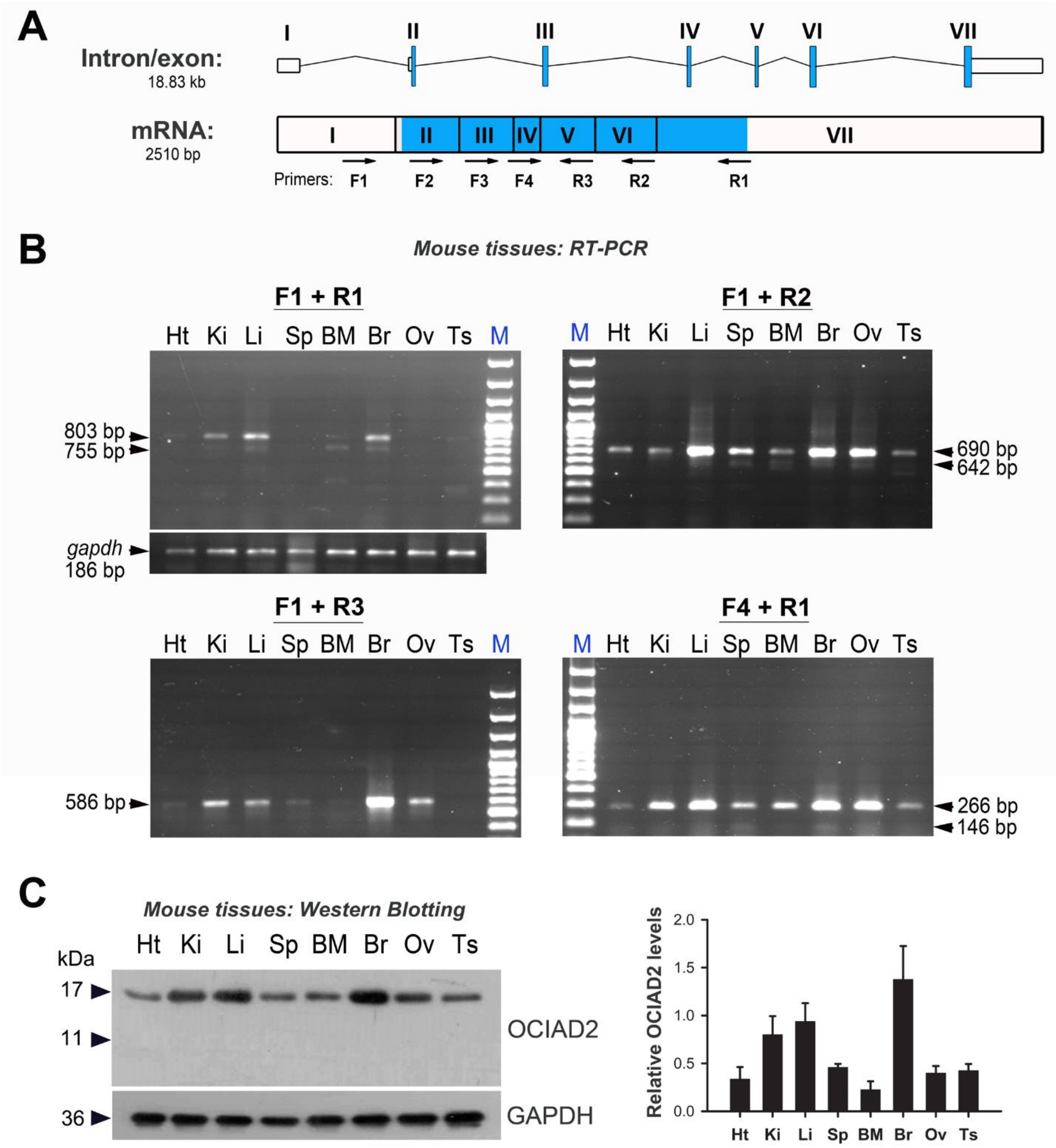

Fig. S3. Analysis of *ociad2* transcript and protein expression in various mouse tissues. (A) Schematic representing the introns (black lines) and exons (boxes) of the mouse *ociad2* gene. Coding regions shown in blue and untranslated regions in white. Exon number indicated in Roman numerals. The arrows below the longest transcript (2510 bp) indicate the position of the forward (F: 1, 2, 3, 4) and reverse (R: 1, 2, 3) primers used for RT-PCRs. **(B)** Differential expression of *ociad2* transcripts. Different combinations of primer pairs (positions identified in **(A)**) were used to perform semi-quantitative RT-PCRs on cDNA from mouse tissues as indicated above the gel pictures. A combination of forward and reverse primers used as indicated to amplify 803 bp, 755 bp (F1 + R1); 690 bp, 642 bp (F1 + R2), 586 bp (F1 + R3) and 266 bp, 146 bp (F1 + R4). **(C)** Western Blotting for detection of OCIAD2 in mouse tissues. Protein size markers (in kDa) are indicated to the left. Tissues used: H, heart; K, kidney; Li, liver; S, spleen; BM, Bone marrow; B, brain; Ov, ovary and Ts, testis. M indicates marker. Data are representative of three independent experiments. Full-length gels and blots are presented in Supplementary Figure S6.

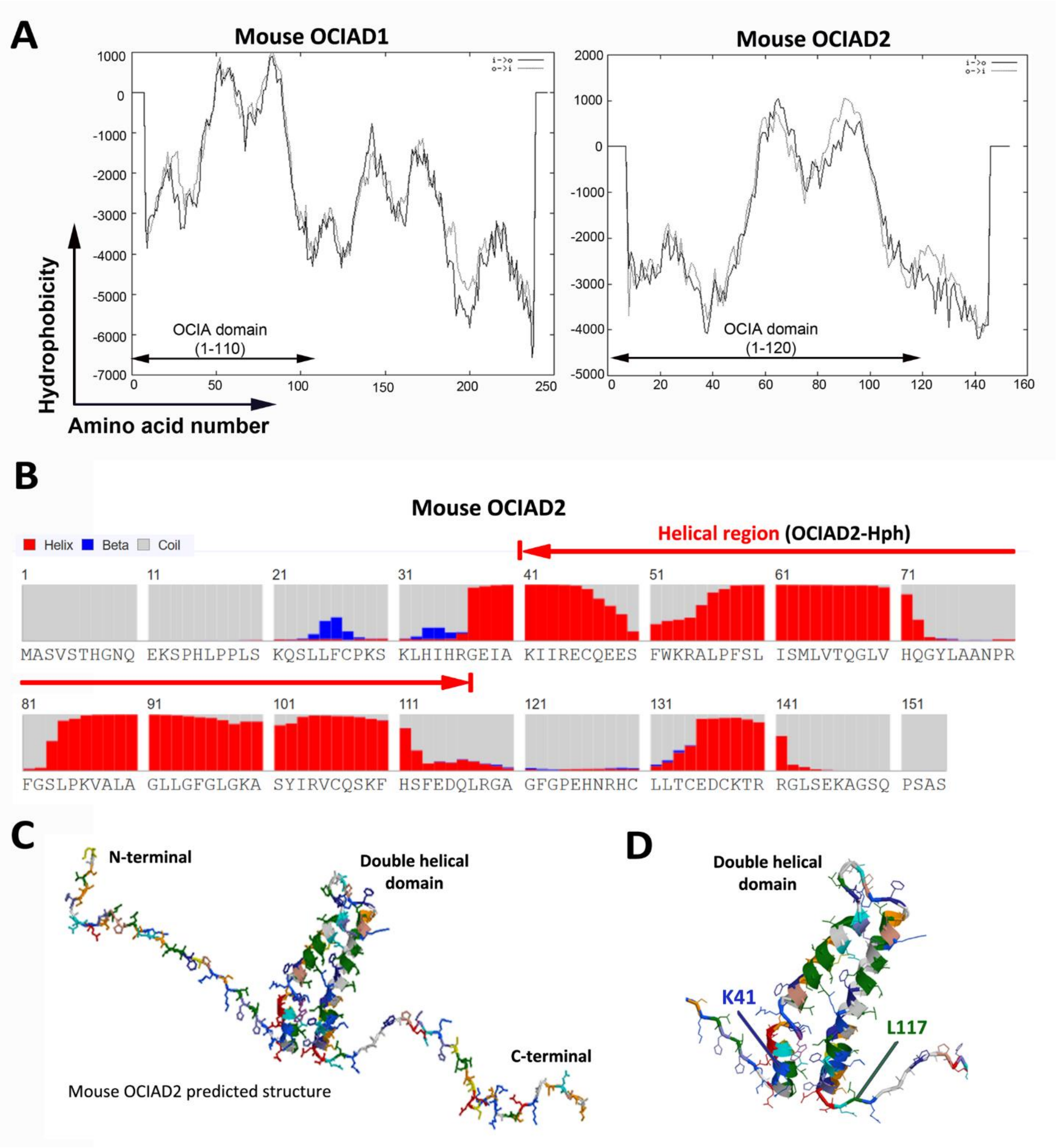

Fig. S4. The central region of OCIAD2 is predicted to contain two alpha helices. (A) Hydrophobicity analysis of mouse OCIAD1 and OCIAD2 (source: TmPred). **(B)** Secondary structure prediction shows that the hydrophobic region of mouse OCIAD2 (mOCIAD2-Hph) is predomi-

nantly helical (source: RaptorX). **(C)** Predicted structure of mouse OCIAD2 showing the N-terminal region, double helical domain and the C-terminal region (source: RaptorX). **(D)** Zoomed view of (C) showing the double helical region ranges from residues phenylalanine 41 to leucine 117.

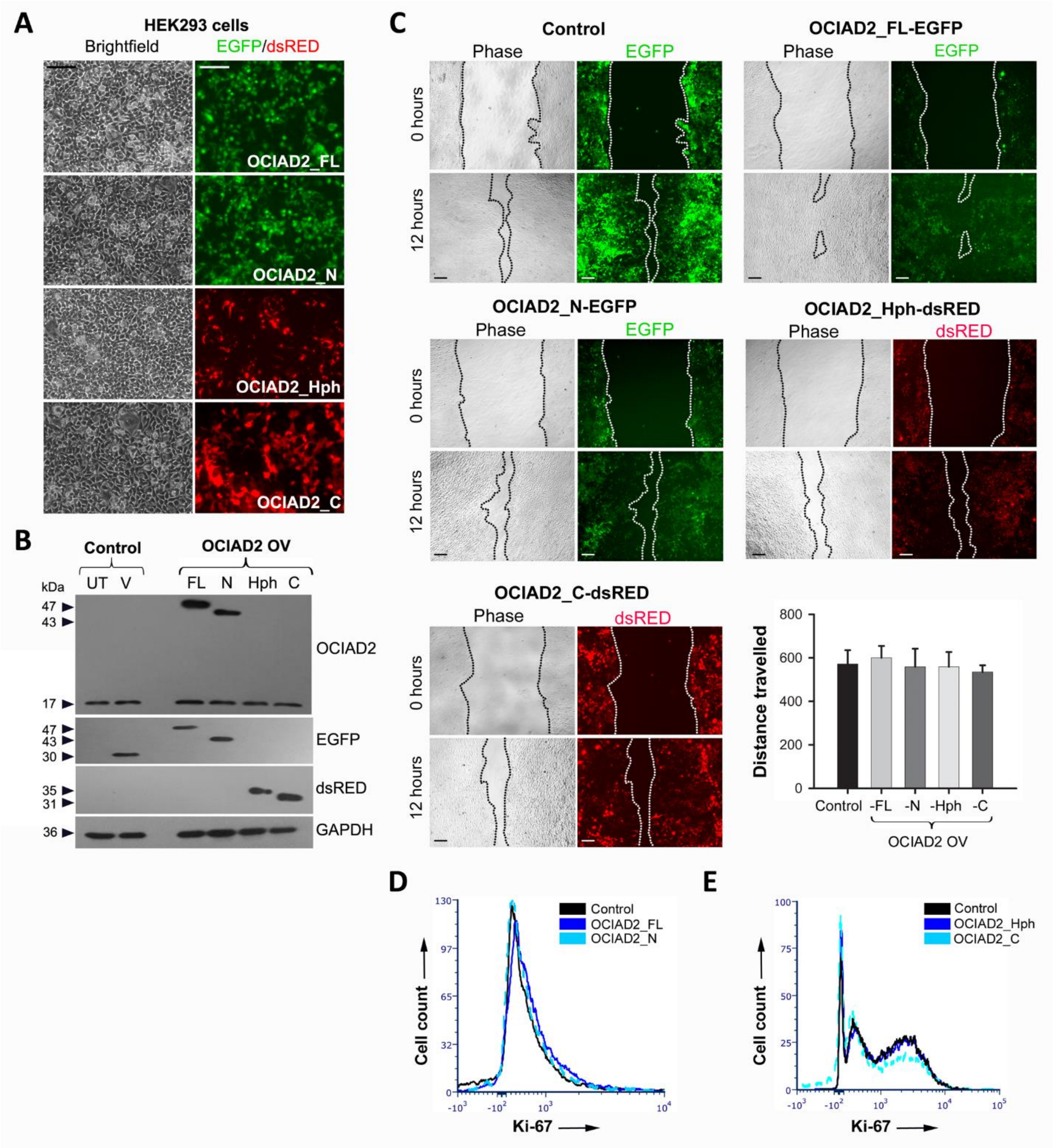

Fig. S5. OCIAD2 overexpression does not influence cell migration or proliferation. (A, B)

Validation of transfection of various OCIAD2 reporter constructs into HEK293 cells by fluorescence imaging and Western Blotting. UT = untransfected, V = empty vector transfected; FL = OCIAD2_FL-EGFP; N = OCIAD2_N-EGFP; Hph = OCIAD2_Hph-dsRED; C = OCIAD2_C-dsRED. Scale bar in (A) is 20 µm. Full-length blots are presented in Supplementary Figure S6. **(C)** No change observed in migration of HEK293 cells overexpressing various OCIAD2 expression constructs. Monolayers of HEK293 cells transfected with empty vector (control), OCIAD2_FL-EGFP, OCIAD2_N-EGFP, OCIAD2_Hph-dsRED and OCIAD2_C-dsRED were scratched and the degree of recovery was measured at 0, 12 and 24 hours post-wounding. Representative phase and fluorescence images shown for 0 and 12 hour time points, 4X magnification. Measurement and estimation of wound recovery was based on the initial wound size. Graph alongside shows quantification of results obtained from three independent experiments (mean $\pm$ SEM). **(D, E)** Flow cytometry analysis of Ki-67$^+$ population in cells overexpressing various OCIAD2 reporter constructs.

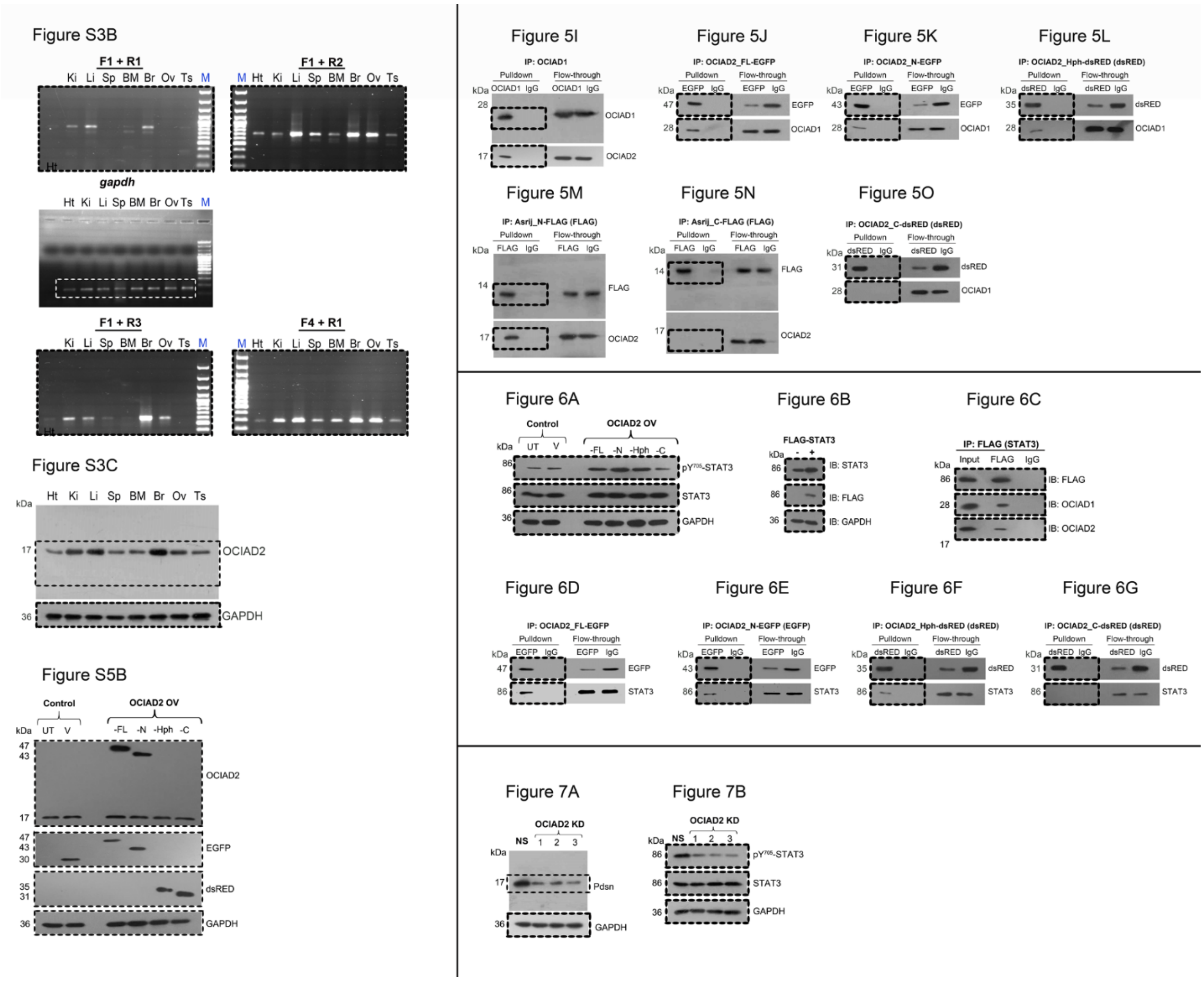

Fig. S6. Uncropped full-length pictures of Western Blotting membranes and DNA gels. Uncropped full-length pictures of Western Blotting membranes and DNA gels presented in the main figures and supplementary figures. Membranes were often cut to enable blotting for multiple antibodies.

Supplementary Table legends

Table S1. List of species and accession IDs of OCIAD proteins used for phylogenetic analysis.

Table S2. Maximum Likelihood fits of 56 different amino acid substitution models. Models with the lowest BIC scores (Bayesian Information Criterion) are considered to describe the substitution pattern the best. For each model, AICc value (Akaike Information Criterion, corrected), Maximum Likelihood value (lnL), and the number of parameters (including branch lengths) are also presented [1]. Non-uniformity of evolutionary rates among sites may be modeled by using a discrete Gamma distribution (+G) with 5 rate categories and by assuming that a certain fraction of sites are evolutionarily invariable (+I). Whenever applicable, estimates of gamma shape parameter and/or the estimated fraction of invariant sites are shown. They are followed by amino acid frequencies (f) and rates of amino acid substitutions (r) for each amino acid pair Relative values of instantaneous r should be considered when evaluating them. For simplicity, sum of r values is made equal to 1 for each model. For estimating ML values, a tree topology was automatically computed. The analysis involved 106 amino acid sequences. All positions with less than 95% site coverage were eliminated. That is, fewer than 5% alignment gaps, missing data, and ambiguous bases were allowed at any position. There were a total of 93 positions in the final dataset. Evolutionary analyses were conducted in MEGA7 [2]. Abbreviations: GTR: General Time Reversible; JTT: Jones-Taylor-Thornton; rtREV: General Reverse Transcriptase; cpREV: General Reversible Chloroplast; mtREV24: General Reversible Mitochondrial.

Supplementary References

1. Nei M. and Kumar S. (2000). Molecular Evolution and Phylogenetics. Oxford University Press, New York.

2. Kumar S., Stecher G., and Tamura K. (2016). MEGA7: Molecular Evolutionary Genetics Analysis version 7.0 for bigger datasets.Molecular Biology and Evolution 33:1870-1874.
